# Inheritance of the CENP-A chromatin domain is spatially and temporally constrained at human centromeres

**DOI:** 10.1186/s13072-016-0071-7

**Published:** 2016-05-31

**Authors:** Justyne E. Ross, Kaitlin Stimpson Woodlief, Beth A. Sullivan

**Affiliations:** Department of Molecular Genetics and Microbiology, Division of Human Genetics, Duke University Medical Center, DUMC, 213 Research Drive, 3054, Durham, NC 27710 USA; Teaching, Learning, and Technology, College of Charleston, JC Long Building, 66 George Street, Charleston, SC 29424 USA

## Abstract

**Background:**

Chromatin containing the histone variant CENP-A (CEN chromatin) exists as an essential domain at every centromere and heritably marks the location of kinetochore assembly. The size of the CEN chromatin domain on alpha satellite DNA in humans has been shown to vary according to underlying array size. However, the average amount of CENP-A reported at human centromeres is largely consistent, implying the genomic extent of CENP-A chromatin domains more likely reflects variations in the number of CENP-A subdomains and/or the density of CENP-A nucleosomes within individual subdomains. Defining the organizational and spatial properties of CEN chromatin would provide insight into centromere inheritance via CENP-A loading in G1 and the dynamics of its distribution between mother and daughter strands during replication.

**Results:**

Using a multi-color protein strategy to detect distinct pools of CENP-A over several cell cycles, we show that nascent CENP-A is equally distributed to sister centromeres. CENP-A distribution is independent of previous or subsequent cell cycles in that centromeres showing disproportionately distributed CENP-A in one cycle can equally divide CENP-A nucleosomes in the next cycle. Furthermore, we show using extended chromatin fibers that maintenance of the CENP-A chromatin domain is achieved by a cycle-specific oscillating pattern of new CENP-A nucleosomes next to existing CENP-A nucleosomes over multiple cell cycles. Finally, we demonstrate that the size of the CENP-A domain does not change throughout the cell cycle and is spatially fixed to a similar location within a given alpha satellite DNA array.

**Conclusions:**

We demonstrate that most human chromosomes share similar patterns of CENP-A loading and distribution and that centromere inheritance is achieved through specific placement of new CENP-A near existing CENP-A as assembly occurs each cell cycle. The loading pattern fixes the location and size of the CENP-A domain on individual chromosomes. These results suggest that spatial and temporal dynamics of CENP-A are important for maintaining centromere identity and genome stability.

**Electronic supplementary material:**

The online version of this article (doi:10.1186/s13072-016-0071-7) contains supplementary material, which is available to authorized users.

## Background

Centromeres are essential chromosomal loci, where kinetochore formation occurs, for segregation of chromosomes in meiosis and mitosis. Human centromeres are composed of alpha satellite DNA with a ~171-bp repeat subunit tandemly organized into higher-order repeat units (HOR) [1–3]. Variability in alpha satellite array size occurs both between chromosomes and among individuals, as repeats of the HORs produce homogenous arrays at each human centromere, ranging from two hundred kilobases up to several megabases in size [4, 5]. However, centromeres are not exclusively dictated by the alpha satellite DNA; specific sequences are neither necessary nor sufficient for centromere formation. Rather, centromere specification is defined by the epigenetic mark of histone variant CENP-A, which replaces canonical H3 in some centromeric nucleosomes [6–9].

CENP-A defines the functional site of a centromere and provides a structural foundation for the kinetochore. Although alpha satellite forms a homogenous array extending over megabases on a given chromosome, centromere proteins normally occupy only a portion of the array [10–13]. This CENP-A/centromeric (CEN) chromatin domain is uniquely arranged as interspersed subdomains of CENP-A and H3 nucleosomes, including both H3.1 and H3.3 [13–17]. Our previous work has shown that the CEN chromatin domain assembles on 30–45 % of any given alpha satellite array [18]. Thus, as with alpha satellite array length, the genomic size of the CENP-A domain varies among non-homologous chromosomes, but is consistently proportional to alpha satellite array size, regardless of chromosome identity. A range of CENP-A domain sizes among human centromeres suggests that the organization of CEN chromatin, such as the concentration of CENP-A nucleosomes within subdomains, varies based on the total genomic extent of the CEN chromatin domain.

In order to maintain genomic stability, the CENP-A domain must be propagated epigenetically over each cell division. Distribution and incorporation of CENP-A have been characterized over the cell cycle, following a pattern dissimilar from canonical histones. Loading of CENP-A is uncoupled from DNA replication, occurring during G1, while during S phase CENP-A is dispersed to newly replicated DNA, and CENP-A protein synthesis occurs only during G2 [19–22]. CENP-A nucleosome numbers have been estimated at ~200 CENP-A nucleosomes that are split into ~100 nucleosomes per centromere during S phase [23]. The manner in which the CENP-A domain is propagated within its genomic context of large, chromosome-specific, alpha satellite arrays has not been determined. The CENP-A domain may expand, contract, or remain static, as CENP-A is loaded and dispersed, depending on how much CENP-A is incorporated each cell cycle and its placement on the alpha satellite array. The number of CENP-A nucleosomes within CEN chromatin changes during replication, and, given the highly homogenous state of alpha satellite, it is not known whether the position of the CENP-A domain or subdomains within CENP-A chromatin are fixed or fluid.

In this study, optical mapping was used to measure the proportion of CENP-A chromatin on the alpha satellite array and determine whether it is fixed in size and location throughout the cell cycle. Additionally, multi-color nascent protein labeling was used to examine the incorporation of new CENP-A over several cell cycles. Our findings indicate that the domain of essential CEN chromatin on each human chromosome is maintained by the location of CENP-A incorporation and dispersal during replication.

## Results

### Similar CENP-A nucleosome quantities are maintained on several chromosomes

**Fig. 1:**
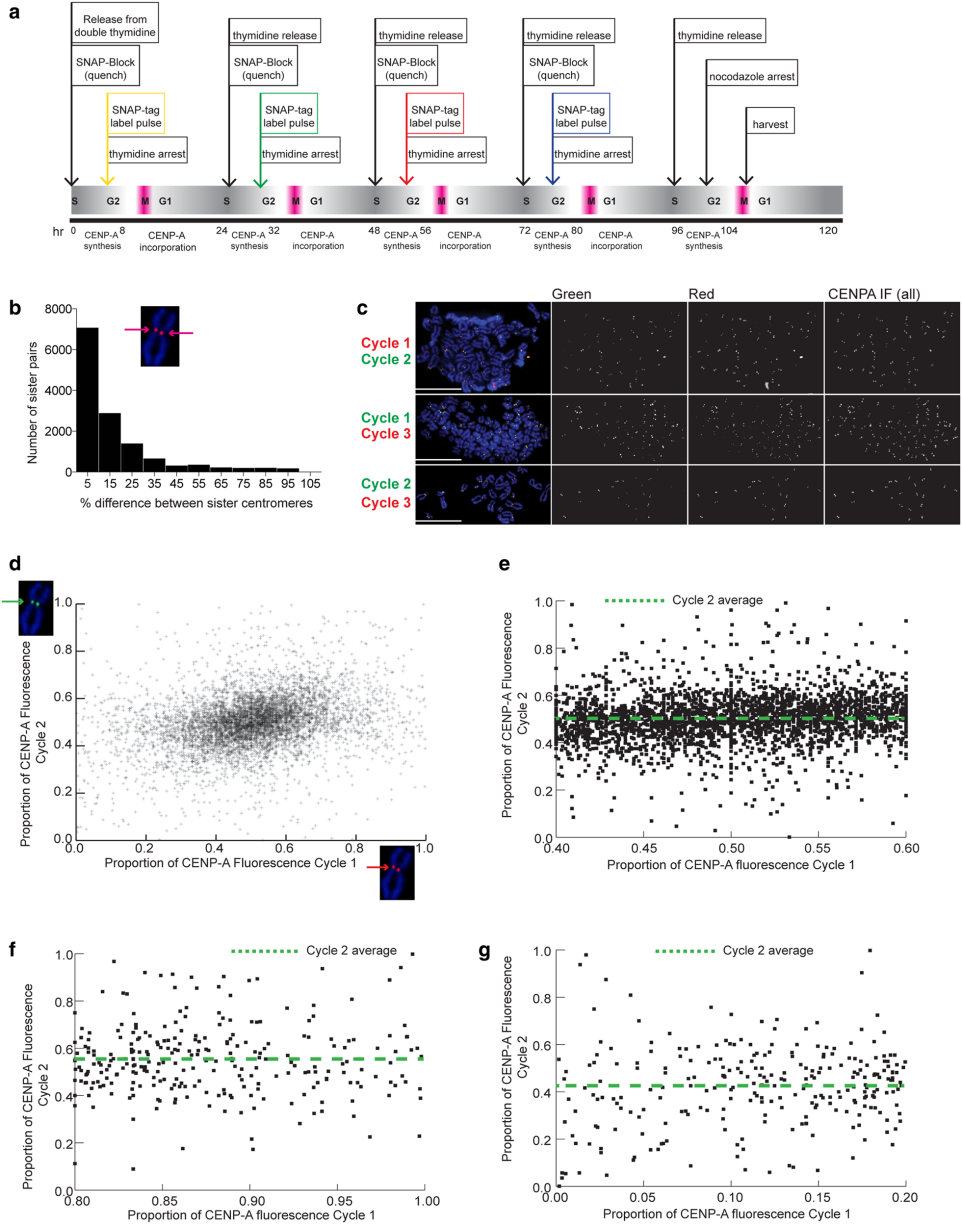
Distribution of distinct pools of nascent CENP-A is equivalent between sister centromeres and between chromosomes. **a** Outline for using quench-pulse-chase experiments to detect distinct nascent pools of SNAP-tagged CENP-A over several cell cycles. After quench-pulse-chase experiments, cells were arrested in metaphase, and total CENP-A was detected by immunostaining with CENP-A antibodies. Quantitative measurements of sister centromeres of individual chromosomes revealed how nascent CENP-A had been distributed in the most recent or previous cell cycles. **b** Frequency distribution of the difference in the total CENP-A intensity (amount of CENP-A) between sister centromeres (*n* > 13,000). **c** Results of multi-labeling of distinct SNAP-CENP-A pools to follow temporal dynamics of CENP-A distribution. Nascent CENP-A from consecutive (Cycle 1 vs Cycle 2; Cycle 2 vs Cycle 3) and alternating cell cycles (Cycle 1 vs Cycle 3) was detected. *Scale bars* 15 μm. **d** Quantitation of CENP-A fluorescence (arbitrary fluorescence units, AFU) at single sister centromeres in two different cell cycles. Chromosome insets highlight nascent CENP-A at single sister centromeres and detected in different cell cycles (*red*, *green arrows*). **e** A subset of the data from **d** showing proportionate CENP-A distribution in Cycle 1 followed by disproportionate distribution in Cycle 2. **f**, **g** Quantitation of SNAP-CENP-A distribution between sister centromeres over different cell cycles, showing the extreme edges (upper 80 % and lower 20 %) of the data set and illustrating disproportionate CENP-A distribution in Cycle 1 followed by proportionate distribution in Cycle 2

Prior studies have suggested that amounts of CENP-A vary among human centromeres and that CENP-A nucleosomes segregate randomly between daughter chromatids during replication [23, 24]. Such variation in CENP-A might be related to alpha satellite array size or could represent normal fluctuation between sister centromeres at individual chromosomes each cell cycle. To more specifically address the S phase distribution of CENP-A over time between sister centromeres and on specific human chromosomes, we labeled and detected nascent CENP-A over multiple cell cycles using SNAP-tag labeling (Fig. 1a). In some experiments, we followed the SNAP labeling with FISH to identify specific human centromeres and track the amount and distribution of CENP-A at the same centromere over multiple cell cycles.

We established an HT1080 cell line expressing SNAP-CENP-A. By Western blotting, we determined that endogenous CENP-A levels were reduced by 26 % (Additional file 1: Figure S1a). SNAP-CENP-A was present at levels similar to endogenous CENP-A within the HT1080-SNAP-CENP-A cell line. It has been shown that exogenous CENP-A competes with endogenous CENP-A, and a normal total level is achieved by down-regulating endogenous CENP-A [19]. If this compensation does not occur, over-expression of CENP-A by 35–70 % over endogenous CENP-A disrupts normal centromeric chromatin dynamics, increasing the size of the CENP-A domain and often leading to neocentromere formation [18, 25, 26]. We measured the size of the CENP-A domain on specific chromosomes in the HT1080 SNAP-CENP-A line and did not observe an increase in CENP-A domain size, an established indicator of altered CENP-A chromatin dynamics (Additional file 1: Figure S1b). We also did not visually observe CENP-A localization elsewhere in the genome, as has been reported for CENP-A over-expressing lines [25, 26]. We conclude that the modestly increased level of CENP-A does not abnormally affect the dynamics of the CENP-A chromatin domain.

HT1080 SNAP-CENP-A lines were arrested in mitosis, since metaphase chromosomes allow analysis of CENP-A distribution during the previous S phase to each sister centromere of individual chromosomes. In each SNAP labeling experiment, we visualized total CENP-A by immunostaining to measure the total amount at each centromere. Nascent CENP-A pools loaded in specific cell cycles were also visualized using different fluorophore-conjugated SNAP-tag labels (TMR-Star and SNAP-Oregon Green) (Fig. 1a). To ensure that we detected distinct SNAP-CENP-A pools, it was necessary to treat cells with SNAP-block (bromothenylpteridine, BTP) each cell cycle to quench SNAP-CENP-A produced in the previous cell cycle (Additional file 1: Figure S1c, d). The fluorescence intensity of total CENP-A and cell cycle-specific pools of SNAP-CENP-A at sister chromatids was measured at all centromeres using a custom imaging script.

We observed that typically the total amount of CENP-A was dispersed evenly between the two sister centromeres; the average for one sister centromere was 50.01 % (±12.83) and 49.99 % (±12.83; *n* = 13,435) for the other (Fig. 1b). SNAP tagging of nascent CENP-A pools loaded into centromeres in consecutive cycles (i.e., Cycle 1 vs Cycle 2) versus alternate (Cycle 1 vs Cycle 3) was informative for comparing the distribution of CENP-A over time (Fig. 1c, Additional file 2: Figure S2b). These experiments revealed that over successive cell cycles, centromere-incorporated CENP-A was, for the most part, split equally between sister centromeres (Fig. 1c, d). Moreover, several data points highlighted that the distribution of CENP-A behaved independently between pools of cycle-specific CENP-A (Fig. 1d). CENP-A could be divided equally in one cell cycle even if the CENP-A from the previous cycle was disproportionately distributed (Fig. 1e–g). For instance, if 85 % of CENP-A was distributed to one sister in cycle 1 (old pool), that same sister did not always receive 15 % of CENP-A in cycle 2 (newer pool). In fact, it was just as likely to receive 50 % of the newer pool of CENP-A (Fig. 1f). Conversely, if the older pool of CENP-A distributed evenly, the newer pool could do so unevenly, suggesting that there was not a bias in the older versus more recently distributed CENP-A pools. These findings also show that we were able to accurately quantitate the amount of CENP-A from older, more dilute CENP-A pools, in addition to the newest pool. Overall, these results indicate a preference for relatively equal distribution during replication of both old and newly incorporated CENP-A each cell cycle and that a previous pattern of unequal CENP-A distribution was not compensated for in the next cell cycle.

**Fig. 2:**
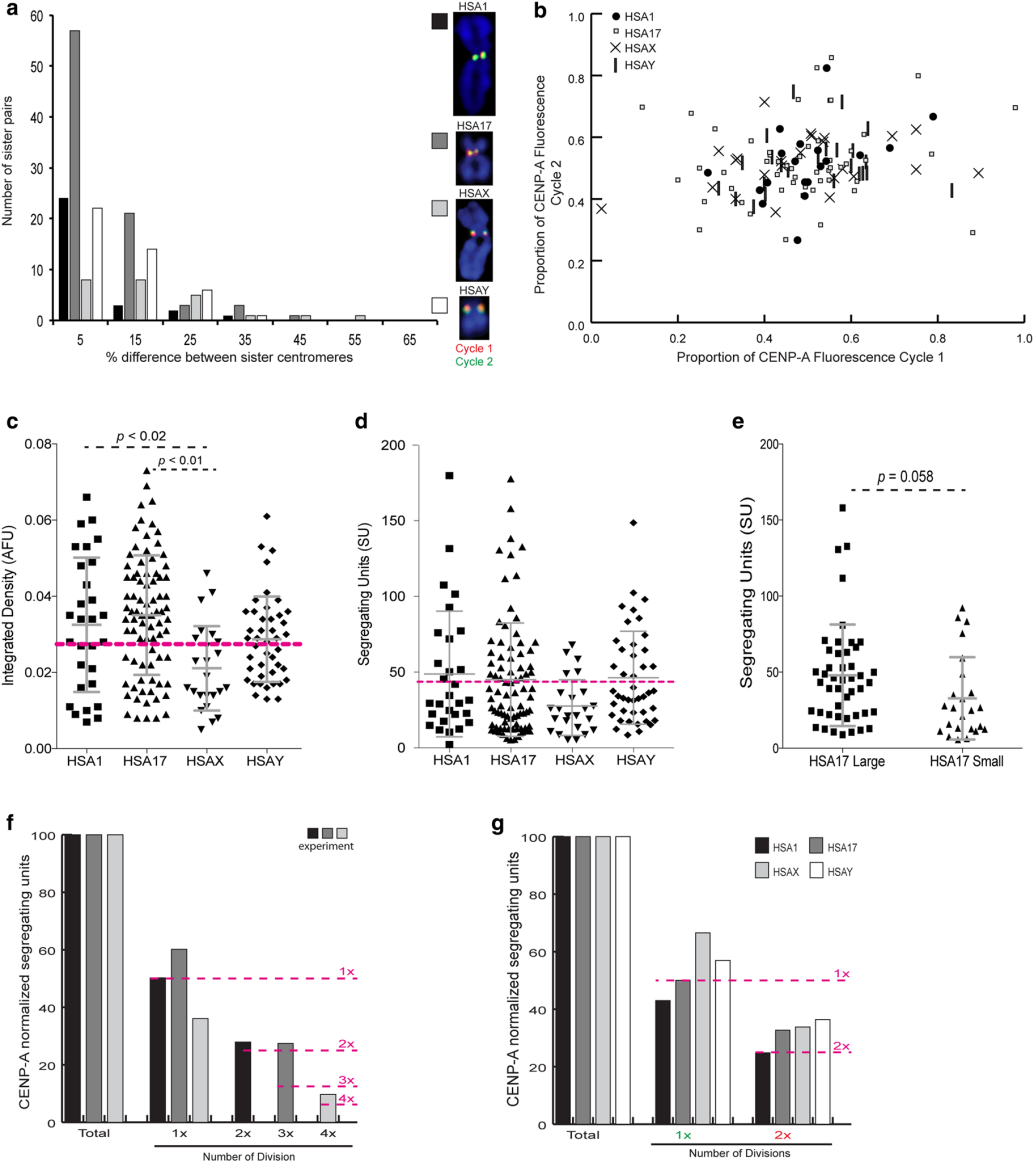
Chromosome-specific dynamics of CENP-A distribution. **a** Frequency distribution of the difference in the total CENP-A intensity (amount of CENP-A) between sister centromeres of chromosomes 1, 17, X, and Y. Nascent CENP-A fluorescence (*green* or *red*) on representative chromosomes represents specific cell cycles (i.e., Cycle 1 and Cycle 2). **b** Quantitation of CENP-A fluorescence (arbitrary fluorescence units, AFU) at single sister centromeres in two different cell cycles for chromosomes 1, 17, X, and Y. **c**, **d** Quantification of total CENP-A fluorescence (**c**) or segregating units (**d**), based on stochastic fluctuation of CENP-A fluorescence, at the centromeres of four human chromosomes. Values for HSA1 and HSA17 represent both chromosomal homologs. The diploid cell line studied was male, so HSAX and HSAY values represent haploid chromosomes. *Dotted pink line* indicates the average across all human centromeres. Mean and standard deviation are indicated for each centromere. **e** The range in CENP-A fluorescence quantified as segregating units (SU) between HSA17 homologs whose alpha satellite arrays vary in size by 2 megabases. Only *p* values <0.01 were considered significant. Mean and standard deviation are indicated. **f** The distribution of nascent CENP-A segregating units normalized to total CENP-A, looking across four different cell cycles. The *pink dotted lines* illustrate dilution of CENP-A over four cell cycles. *Bars* represent data from three different experiments, each of which included two of four nascent CENP-A labelings, i.e., Cycles 1 and 2 (*black bars*), Cycles 1 and 3 (*medium gray bars*), and Cycles 1 and 4 (*light gray bars*). **g** The distribution of nascent CENP-A segregating units normalized to total CENP-A, looking across two different cell cycles at the centromeres of specific human chromosomes. *Each bar* represents the centromere of a single chromosome. *Dotted pink line* indicates the dilution of CENP-A over two cell cycles

We also specifically tracked CENP-A (total and discrete pools) at the centromeres of *Homo sapiens* chromosomes 1, 17, X, and Y (HSA1, HSA17, HSAX, HSAY). These chromosomes were chosen because they represent a range in chromosome and alpha satellite sizes (large arrays: both HSA1s, HSAX, one HSA17; small: one HSA17, HSAY) [18, 27]. As observed for all chromosomes, for these four chromosomes, CENP-A was evenly divided between the sister centromeres over multiple cell cycles (Fig. 2a, b). These data suggest that the inheritance patterns of CENP-A at each centromere of different chromosomes are similar. Our results also suggest that CENP-A does not covary drastically between sister centromeres. In the human cell line we analyzed, nascent CENP-A was equally segregated to daughter chromatids during S phase, regardless of chromosome identity or alpha satellite array size.

Alpha satellite array sizes can vary two- to threefold among chromosomes and between homologs. Likewise, CENP-A chromatin domain size varies in the total genomic distance that it occupies [18]. These findings suggest that centromeres contain varying amounts of either CENP-A or CENP-A nucleosomes are positioned differently along a short versus long alpha satellite array. To test the former possibility that large centromeres retain more CENP-A than small centromeres, we focused on four chromosomes whose alpha satellite arrays ranged in size (Fig. 2). The different centromeres exhibited similar amounts of total CENP-A, near the average for all centromeres (0.0137 ± 0.00746 AFU) (Fig. 2c). However, a one-way analysis of variance did reveal a difference in the levels of CENP-A fluorescence among the individual chromosomes we studied [*F*(3, 176) = 3.546, *p* = 0.0004]. Post hoc comparisons using the Tukey HSD test indicated that HSAX differed significantly from HSA1 and HSA17 (*p* values of <0.02, and <0.01, respectively) (Fig. 2c, Additional file 3). However, all other pairwise comparisons (between HSA1, HSA17, and HSAY) were insignificant (*p* values ≥0.1).

Although we observed that nascent CENP-A pools were largely divided equivalently between sisters, we took advantage of the fact that slight deviations from exact halving of CENP-A fluorescence intensity on sister centromeres can be used to calculate the number of heritable segregating units (SUs) of CENP-A at each centromere [23, 28]. As defined previously by other groups, SUs are approximated from measurements of normalized fluorescence intensities (in this case, distinctly labeled total CENP-A or SNAP-CENP-A pools) at each sister centromere [23, 29]. We calculated that each sister centromere had an average of 43.65 (±47.36) SUs of CENP-A (Fig. 2d). The number of SUs of CENP-A from each pool of SNAP-CENP-A reflected the number of divisions for which that pool of CENP-A had been incorporated at the centromere of a given chromosome (Fig. 2f, g). Each SU consists of at least one nucleosome that contains two molecules of CENP-A [23, 29]; however, CENP-A nucleosomes can segregate in multi-nucleosome units. Thus, our calculation of approximately 50 SUs per chromatid corresponds to a conservative estimate of a minimum of 100 CENP-A molecules per centromere.

When comparing specific chromosomes, even though they had different alpha satellite array sizes, we found no significant differences in segregating units of CENP-A (Fig. 2d). A lower level of fluorescence at HSAX suggested that HSAX in this cell line contained less total CENP-A; indeed, it did have the fewest number of calculated segregating units, although it was not a statistically significant difference from the other chromosomes or the overall average. This particular HSAX had an average-sized (3 Mb) alpha satellite array [18], so the reduced number of CENP-A molecules and CENP-A SUs may represent the lower limit of CEN chromatin that a centromere can have while still remaining stable. Alternatively, the sex chromosomes may be more flexible in the amount of CENP-A at their centromeres, since previous studies reported that the Y centromere had less CENP-A overall [23, 24]. In our cell line, HSAY did not exhibit less CENP-A overall, but was similar to HSA1 and HSA17 (Fig. 2c–d).

While all chromosomes exhibited equivalent segregating units of CENP-A, we noted a wider range of SUs for HSA1 and HSA17 (Fig. 2d) and hypothesized that it could be due to variation in alpha satellite array sizes between homologs since the HT1080 cell line contained more than one HSA1 and HSA17 chromosome. To address this possibility, we analyzed each HSA17 independently, since the two-megabase difference in the sizes of their alpha satellite arrays makes them easily distinguishable by FISH (ME Aldrup-MacDonald et al., unpublished data). The smaller HSA17 trended toward slightly fewer SUs, but did not reach statistical significance (*p* = 0.058) (Fig. 2e). Overall, then, our data indicate that SUs of CENP-A are comparable between different chromosomes and even different homologs of the same chromosome, agreeing with previous studies indicating that human chromosomes contain similar heritable units of CENP-A [23]. Varying CENP-A chromatin domain sizes that have been measured on chromatin fibers may instead reflect differences in the density of CENP-A nucleosomes within the subdomains that make up the entire CEN domain. Collectively, this set of SNAP experiments indicates that in HT1080 cells, the total, heritable amount of CENP-A does not vary significantly among chromosomes.

### The CENP-A domain exists on a subregion of the alpha satellite DNA array throughout the cell cycle

Previous studies have shown that CENP-A is usually assembled on a portion of alpha satellite DNA in human cells [10, 11, 30]. Our own studies have established that the genomic size of the CENP-A chromatin domain is consistently proportional to total alpha satellite array size, ranging from 30 to 45 % of the array [18]. However, it is not clear how this proportion is affected by the cell cycle dynamics of CENP-A loading and dispersal at individual centromeres. During each cell cycle, CENP-A is loaded onto alpha satellite DNA in late mitosis/early G1, replacing placeholder H3.3, and is then diluted during replication in S phase [16, 19]. It is not known whether CENP-A is loaded along the entire alpha satellite array in G1 and is diluted in S phase to produce a limited domain of CENP-A chromatin or whether a defined domain of CENP-A is present throughout the cell cycle and CENP-A is loaded and diluted within this confined domain. To address whether the domain expands and contracts within the alpha satellite array as new CENP-A is incorporated and dispersed each cell cycle, we investigated the size of the CENP-A domain throughout the cell cycle.

Stretched chromatin fibers were prepared from synchronized cells to simultaneously visualize specific alpha satellite arrays and CENP-A. We focused on the alpha satellite arrays of chromosome X (DXZ1) and chromosome Y (DYZ3) in three male cell lines (Additional file 4). These chromosomes were chosen since they are haploid and allow unequivocal assignment of an alpha satellite array and CENP-A domain to a specific chromosome [18]. CENP-A occupancy was defined at three time points in the cell cycle: during G1 phase, at the G1/S phase boundary, and during S phase (Additional file 5: Figure S3a). The thymidine analog EdU was used in each experiment and was detected using Click-iT chemistry prior to CENP-A immunostaining-FISH. Only EdU-negative fibers were analyzed in the G1 and G1/S experiments; conversely, only EdU-positive fibers were analyzed from the S phase synchronizations to ensure that only centromeres that had replicated were measured (Additional file 5: Figure S3b).

**Fig. 3:**
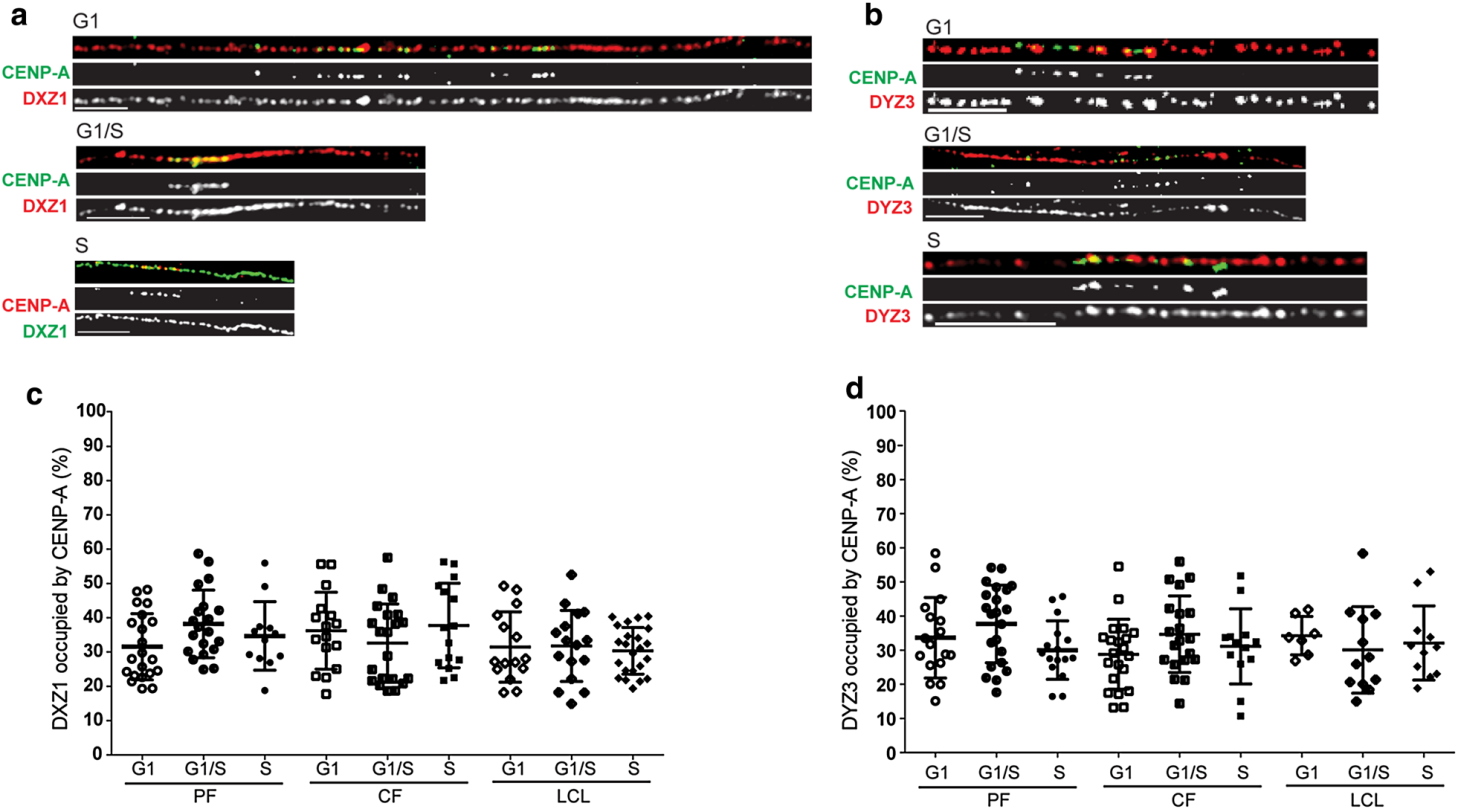
The CENP-A domain occupies a similar proportion of alpha satellite DNA throughout the cell cycle. Chromatin fibers were isolated from three male cell lines that were synchronized in G1, G1/S, and S phase. Fibers were immunostained for CENP-A, followed by hybridization with alpha satellite probes to the centromeres of HSAX (DXZ1) or HSAY (DYZ3). Each alpha satellite array varied in length, as previously determined by molecular analyses [18]. **a**, **b** Results of chromatin fiber CENP-A immunostaining (*green*) and alpha satellite FISH (*red*). *Scale bars* are 5 μm. **c**, **d** Percentage of alpha satellite occupied by CENP-A chromatin throughout the cell cycle. The lengths of the FISH signal and CENP-A signal were measured, and the proportion of the alpha satellite array occupied by CENP-A chromatin was calculated for each cell cycle stage. *PF* primary fibroblast, *CF* cancer fibroblast, *LCL* lymphoblastoid cell line

CENP-A domains on DXZ1 (CENP-A^DXZ1^) within the three cell lines varied from 0.47 Mb (±0.13 Mb) to 1.53 Mb (±0.50 Mb). However, at all time points, CENP-A^DXZ1^ domains assembled on approximately one-third (33 ± 11 %) of the alpha satellite array, regardless of total size (Fig. 3a, c). Overall, the CENP-A^DXZ1^ domain remained consistently present on approximately one-third of the alpha satellite array from G1 phase to S phase (Fig. 3c, Additional file 4).

Similarly, CENP-A domain sizes on DYZ3 (CENP-A^DYZ3^) were constant across the cell cycle on individual fibers from the same line. The CENP-A domain in each line was assembled on a similar proportion of the alpha satellite array (34 ± 13 %) (Fig. 3b, Additional file 4), corresponding to consistent assembly of CENP-A on approximately one-third of DYZ3 across the cell cycle (Fig. 3d). Taken together, these results provide strong evidence that CENP-A chromatin domains are proportionally assembled and maintained throughout the cell cycle on about one-third of an alpha satellite array.

### The CENP-A domain is consistently assembled at the same position within the alpha satellite array

We have presented strong evidence that the CENP-A domain size remains proportional to alpha satellite array size throughout the cell cycle, but the dynamics of the finer-scale spatial positioning of the CENP-A domain have not been determined. It is not known whether the CEN chromatin domain is fixed or shifts across the many megabases of alpha satellite DNA at human centromeres. For instance, the CENP-A domain could move to a new location within the alpha satellite array each cell cycle or its position on the DNA might differ from cell to cell. Because alpha satellite arrays are extremely homogenous at the DNA level [31], sequences within the arrays cannot be used as landmarks. Instead, we used two single molecule optical mapping approaches to test whether CENP-A domain position is static or fluid on alpha satellite DNA in a population of cells.

**Fig. 4:**
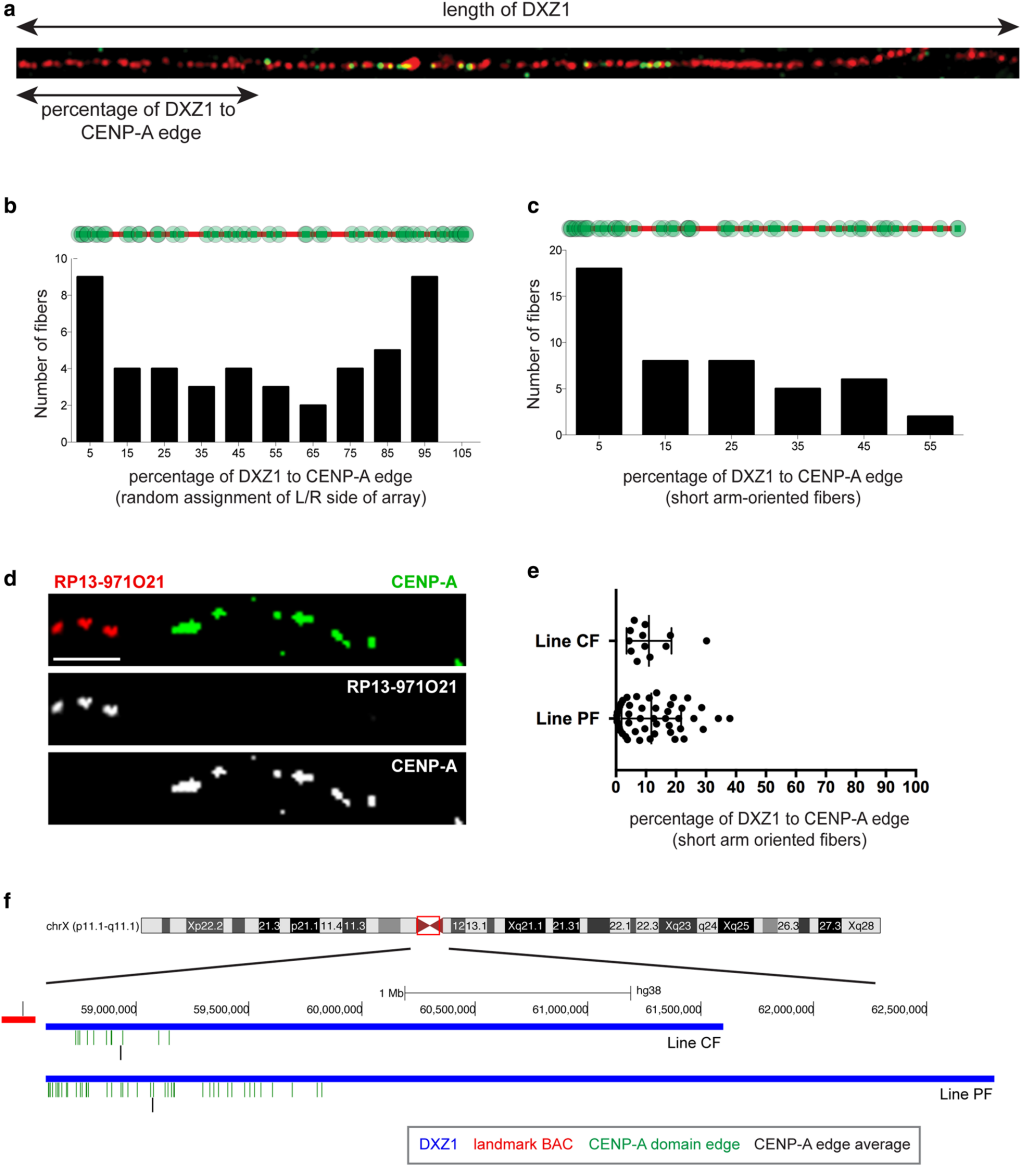
The CENP-A domain is inherited at the same overall location on alpha satellite DNA. **a** Representative chromatin fiber showing CENP-A immunostaining (*green*) and FISH with an alpha satellite probe to DXZ1 (*red*). The distance between the edges of DXZ1 and the edge of the CENPA domain was measured for each fiber. **b** Calculation of the percentage of DXZ1 between its edge and the edge of the CENP-A domain. For this analysis, the orientation of the alpha satellite array (short arm vs long arm of HSAX) was not known. The direction of each fiber was randomly assigned such that half of the fibers were oriented with CENP-A domains closer to the arbitrary left end of the DXZ1 FISH and half were oriented with CENP-A closer to the arbitrarily assigned right edge of the DXZ1 fluorescent signal. **c** Analysis of CENP-A domain skewness, by non-randomly assigning the shortest distance between the DXZ1 edge and the leading edge of the CENP-A immunostaining as orientation toward the HSAX short arm. This assumption was made based on previous studies suggesting that centromere proteins are biased toward the short arm side of DXZ1 [11, 52]. **d** Representative chromatin fiber IF-FISH image showing the position of CENP-A immunostaining (*green*) relative to the proximal short arm landmark BAC RP13-971O21 (*red*) located adjacent to DXZ1, the alpha satellite array on the human X chromosome. *Scale bar* is 5 μm. **e** Measurement of the percentage of DXZ1 between the short arm edge of the array and the edge of the CENP-A domain in two different cell lines. **f** BAC RP13-971O21 (*red line*) was used as a landmark representing the short arm side of the DXZ1 array. Genomic distances representing the placement of the left edge of the CENP-A domain relative to the short arm edge of DXZ1 (*blue line*) on individual chromatin fibers (*green ticks*) are shown as custom tracks in the UCSC genome browser illustrating the non-random positioning of the CENP-A domain on the short arm side of the DXZ1 array (*blue line*) for two different cell lines. The average starting edge of the CENP-A domain based on the distance from the short arm edge of DXZ1 is shown as a *single black tick* under the individual fiber data

Stretched chromatin fibers that had been immunostained for CENP-A and hybridized with alpha satellite probes were analyzed for positional bias of the CENP-A domain within the DXZ1 array (HSAX centromere). On these individual chromatin fibers, the orientation of the alpha satellite array with respect to the HSAX chromosome arms (short vs long) was not known. Therefore, in our first analyses (*n* = 47 fibers), the orientation of each fiber was randomly assigned so that half had CENP-A domains closer to the arbitrary left edge of the DXZ1 FISH signal and half had CENP-A domains closer to the arbitrarily assigned right edge of the DXZ1 fluorescent signal. The distance between the edge of the alpha satellite fluorescent signal and the edge of the CENP-A domain was measured (Fig. 4a). This broad analysis revealed that the CENP-A domain was more frequently located near an end of the DXZ1 array rather than in the middle of the array (Fig. 4b). These experiments implied that the CENP-A/CEN chromatin domain is fixed and non-randomly positioned on alpha satellite DNA and prompted further investigation of the precise location of the domain.

Previous studies of mechanically stretched metaphase chromosomes suggested a bias for centromere protein assembly closer to the short arm side of DXZ1 [11, 32]. When the CENP-A immunostained fibers were oriented with this in mind, the CENP-A domain could be further positioned to determine the degree of asymmetry in placement along the alpha satellite array. Our analyses showed that the CENP-A domain was moderately skewed, favoring a position that was 18.8 % (±15.6) from the edge of the alpha satellite array (0.50 Fisher–Pearson coefficient of skewness) (Fig. 4c).

These analyses, while compelling, still did not permit unequivocal discrimination between the short arm versus long arm side of the DXZ1 array. Our second approach incorporated known genomic markers, or landmarks, outside the DXZ1 array to establish the location of the CENP-A domain relative to the short arm. Chromatin fibers were simultaneously immunostained for CENP-A and hybridized with a BAC probe (RP13-971O21, accession AL591645) located within the pericentromere region immediately adjacent to DXZ1 on the proximal short arm [32]. An additional BAC probe (RP13-775K13, accession AL512884) located distal to RP13-971O21 was used in some experiments to clarify orientation of RP13-971O21. The CENP-A domain was consistently located near the short arm side of DXZ1 closer to the landmark BAC (Fig. 4d). We then measured the distance between the short arm edge of DXZ1 and the edge of the CENP-A domain in two different cell lines (lines PF and CF). Using the fluorescent signal for CENP-A^DXZ1^ and its known size in each line as a conversion factor, we calculated that the edge of the CENP-A domain in line CF was located 329 kb (±225 kb) from the edge of the DXZ1 array. Line PF has a slightly larger DXZ1 array, so that the short arm edge of its CENP-A domain was positioned 459 kb (±365 kb) from the short arm edge of DXZ1 (Fig. 4f). Nevertheless, despite the DXZ1 total array size difference between the two lines, we found that ~10 % of the DXZ1 array was present between the short arm edge of the array and the edge of the CENP-A domain, suggesting a non-random position for centromeric chromatin (Fig. 4e). Our data strongly indicate that the CENP-A domain is assembled at a similar location within DXZ1. Our data raise the possibility that structural or genomic features of DXZ1 or within the pericentromere may influence the consistent position of CENP-A chromatin assembly.

### Nascent CENP-A molecules are incorporated within the existing CENP-A domain in a cycle-specific oscillating pattern

**Fig. 5:**
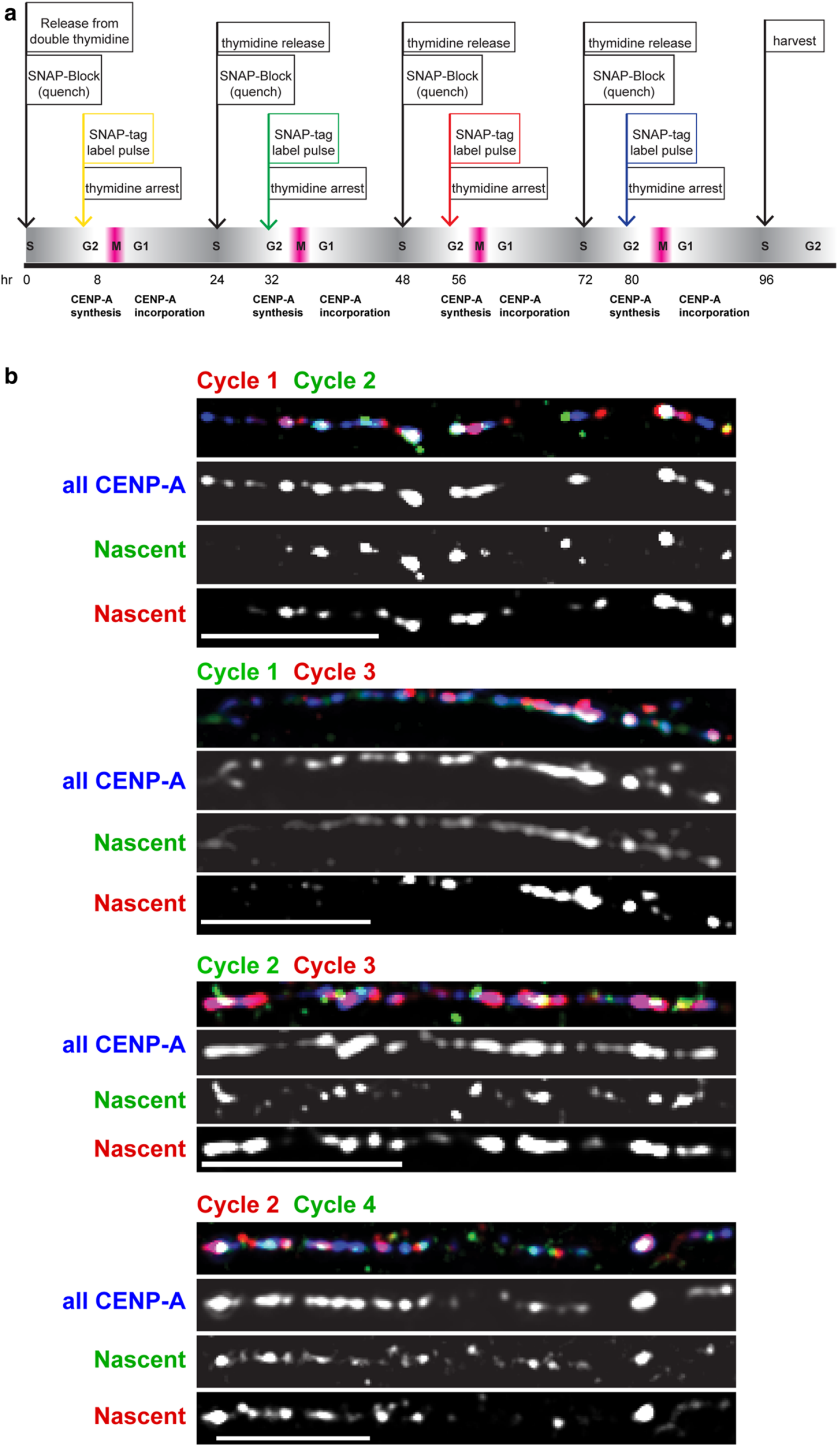
Visualization of spatial positioning of nascent CENP-A on chromatin fibers. **a** Experimental outline of multi-color labeling of nascent CENP-A pools from distinct cell cycles. **b** Representative chromatin fiber images showing total CENP-A (*blue*) and nascent CENP-A pools loaded in consecutive or alternate cell cycles. *Scale bars* are 5 μm

These unexpected results prompted us to investigate how the CENP-A domain is maintained as a limited domain at the same location on the alpha satellite DNA. CENP-A loading occurs in early G1 phase [19] and replaces placeholder H3.3-containing nucleosomes that fill the gaps created by CENP-A distribution during replication [16]. We hypothesized that CENP-A loading occurs primarily within the existing CEN chromatin domain in order to maintain its largely static position on alpha satellite DNA. To test this, we monitored the spatial positioning of nascent CENP-A molecules by leveraging our ability to track CENP-A loading on chromatin fibers over multiple cell cycles. In this approach, we visualized total CENP-A by immunostaining and nascent CENP-A by SNAP-tag labeling, in combinations of two cell cycles across a total of four cell cycles (Fig. 5, Additional file 6: Figure S4a, S4b). Total and nascent CENP-A location was analyzed across chromatin fibers using fluorescent line plots (Additional file 6: Figure S4c) and customized correlation coefficient and object-based mass-particle coincidence computer scripts (Fig. 4).

**Fig. 6:**
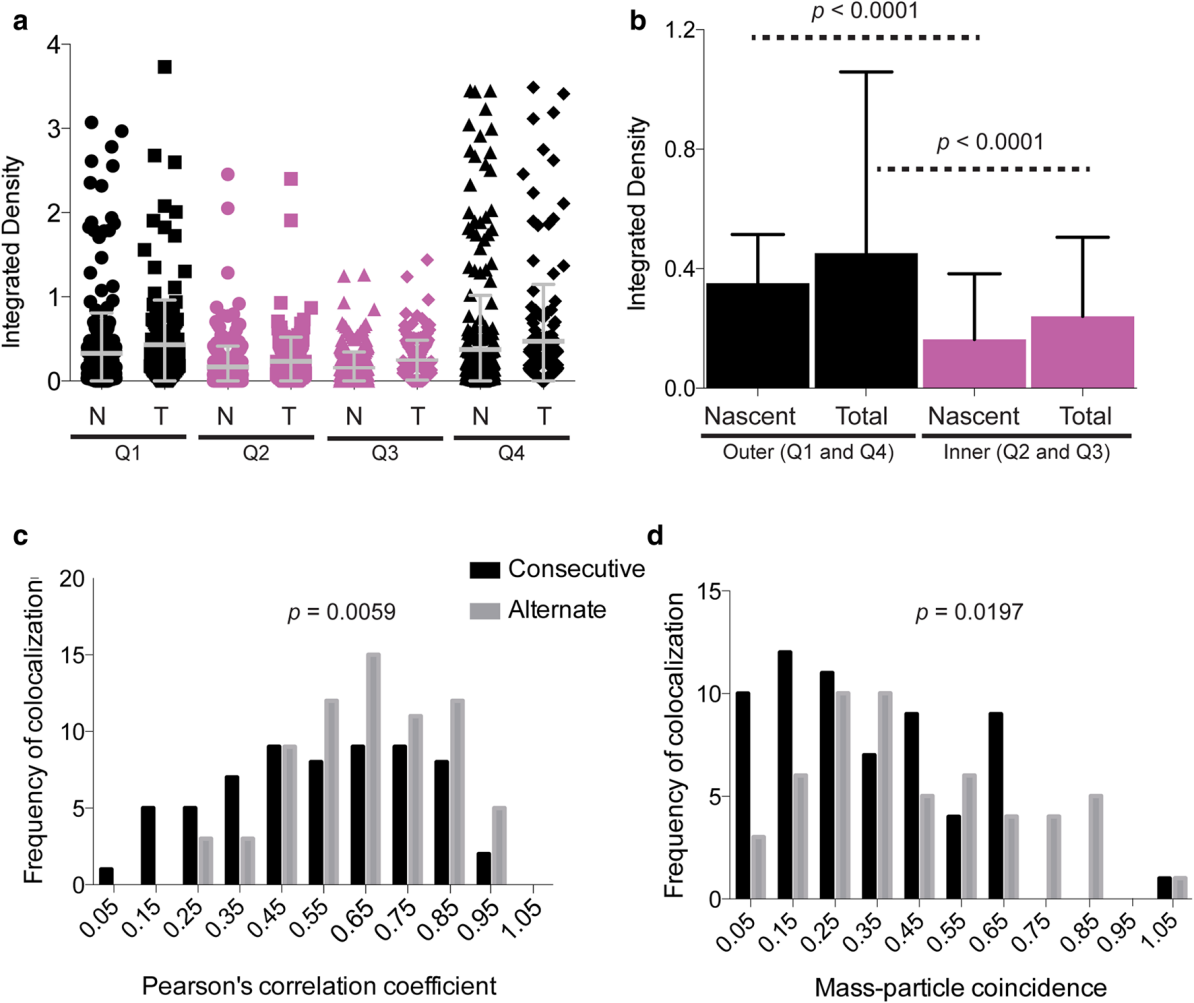
Nascent CENP-A is distributed throughout the existing CENP-A domain, with a bias toward the edges. **a** Quantitation of nascent (*N*) and total (*T*) CENP-A fluorescence on single chromatin fibers divided into four equal quarters. **b** Distribution of CENP-A loading into the outermost versus innermost quarters of the CEN chromatin domain. **c** The Pearson coefficient of co-localization was used to compare positioning of nascent fluorescent CENP-A pools within the CEN chromatin domain in consecutive versus alternate cell cycles. A coefficient of 1 indicates perfect co-localization. **d** Quantification of nascent CENP-A loading in consecutive versus alternate cycles using object-based mass-particle coincidence. A higher value indicates stronger co-localization of CENP-A fluorescence from distinct nascent protein pools

We observed that nascent CENP-A molecules in each cell cycle were loaded at positions within the original CENP-A domain and that new CENP-A loading did not move the domain or create a new domain elsewhere every cycle (Fig. 5b, Additional file 6: Figure S4). In addition, little detectable nascent CENP-A was loaded outside of the established CENP-A domain (Additional file 7: Figure S5a). If fibers were divided into four equal quarters, the majority showed that CENP-A loading occurred across the entire CENP-A domain (Fig. 6a, Additional file 8). However, a larger proportion of CENP-A was incorporated in the outer quarters compared with the inner quarters, corresponding to a preference for nascent CENP-A loading near the edges of the domain (Fig. 6a, b and Additional file 8 for fluorescence quantitation of nascent CENP-A on chromatin fibers). Normalizing the nascent CENP-A levels by the total CENP-A levels revealed that there was in fact an overabundance of nascent CENP-A toward the outside of the domain compared with the inside (*p* < 0.0001; Fig. 6b, Additional file 7: Figure S5b and S5c, Additional file 8).

Labeling distinct pools of nascent CENP-A allowed us to compare loading of CENP-A produced in consecutive cycles (e.g., Cycle 1 vs Cycle 2) versus alternating cycles (e.g., Cycle 1 vs Cycle 3). We measured the intensity of overall fluorescent signal overlap between consecutive and alternate pools of nascent CENP-A using Pearson’s correlation coefficient. CENP-A loaded in consecutive cycles overlapped less than CENP-A loaded in alternate cycles [0.548 ± 0.228 (*n* = 63) vs 0.648 ± 0.186 (*n* = 70; *p* = 0.0059), respectively] (Fig. 6c). When we measured fluorescent signal positions by an alternative method, i.e., mass-particle coincidence, we observed a similar trend, in that CENP-A from consecutive cycles was less coincident (32 ± 23 %; *n* = 63) than CENP-A from alternate cycles (42 ± 25 %; *n* = 54) (*p* = 0.0197) (Fig. 6d). Collectively, the chromatin fiber experiments revealed for the first time the dynamics of CENP-A loading over time. Nascent CENP-A is loaded in an oscillating pattern primarily within the existing CENP-A domain, and more CENP-A is loaded at the edges rather than in the center of the domain. Notably, the chromatin fibers were not marked for specific centromeres, so our experiments captured the global dynamics of CEN (CENP-A) chromatin domains at all human centromeres. Our findings suggest that the pattern of new CENP-A loading may be responsible for restricting the CEN chromatin domain to a finite size and to a specific region of the alpha satellite DNA.

## Discussion

In this work, we have addressed several aspects of CENP-A and CEN chromatin dynamics, including the temporal distribution of CENP-A at individual centromeres, the placement of CEN chromatin with respect to alpha satellite DNA, and the loading dynamics of CENP-A within the defined CEN chromatin domain. This work relied on analysis of sister centromeres at metaphase to reflect the dispersion of CENP-A nucleosomes during S phase. We cannot formally discount the possibility that CENP-A is unequally distributed in S phase and subsequently reorganized to an equal distribution, but as yet no studies exist to substantiate this model. Overall, our findings highlight particular patterns of CENP-A loading and distribution at all human centromeres and suggest that CEN chromatin dynamics at human centromeres is broadly similar. Our results also raise several questions that can be addressed in future studies.

## CENP-A distribution and abundance on different chromosomes

**Fig. 7:**
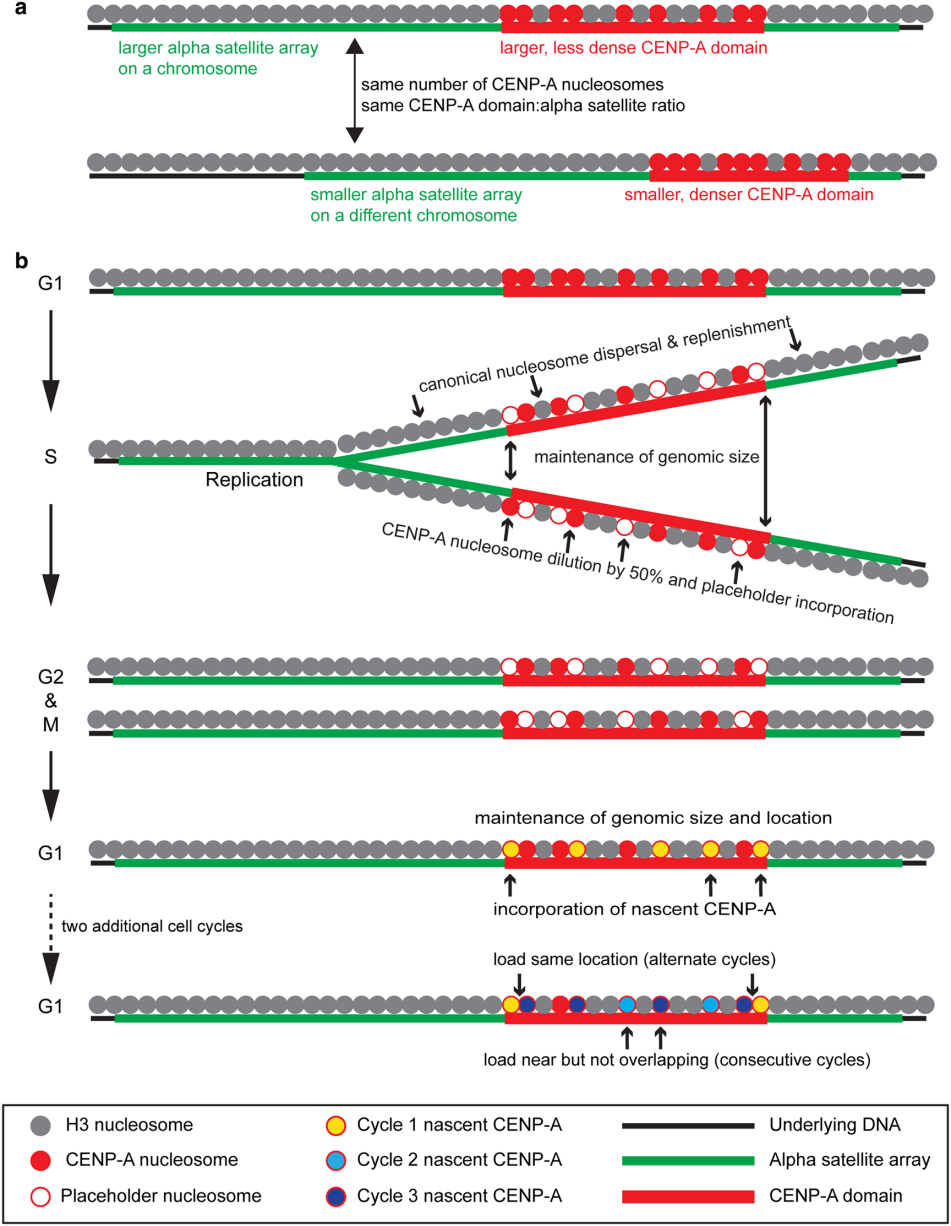
Model for loading and distributing CENP-A nucleosomes in order to retain chromosome-specific CENP-A chromatin domains. **a** Chromosomes with distinct alpha satellite arrays sizes (*green line*) contain similar amounts of CENP-A (*red circles*), most likely because CENP-A nucleosome density within CEN chromatin (*red line*) varies between small and large alpha satellite arrays. The CEN chromatin domains are proportional (30–45 %) to alpha satellite array size. **b** During replication, nascent CENP-A nucleosomes are dispersed equally between mother and daughter strands, and placeholder nucleosomes (*white circles*) containing H3.3 fill the gaps left by CENP-A dilution. In consecutive cell cycles, new CENP-A (*yellow circles*) is placed near old (*red*) CENP-A nucleosomes within the CEN chromatin domain, replacing H3.3 nucleosomes. In the second (consecutive) cell cycle, new CENP-A (*light blue circles*) is loaded near but not overlapping with Cycle 1 CENP-A (*yellow circles*). In the third (alternating) cell cycle, new CENP-A (*dark blue circles*) is loaded near or at the same location as older, Cycle 1 CENP-A, and near, but not overlapping with, Cycle 2 CENP-A (*light blue circles*)

Analyzing specific chromosomes with varying sizes of alpha satellite arrays (HSA1, HSA17, and HSAY) showed that the amount of CENP-A did not differ significantly from the overall average. This finding agrees with previous studies indicating that human centromeres generally contain similar numbers of CENP-A molecules [23]. Chromatin fiber studies of mammalian and *Drosophila* centromeres have shown that CENP-A chromatin only localizes on a portion of the satellite DNA [12, 18, 33], suggesting that limiting CEN chromatin to a region of repetitive DNA is a common feature of endogenous metazoan centromeres. At human centromeres, the CENP-A chromatin domains are proportional to the underlying alpha satellite array sizes that can range by twofold between homologous chromosomes and by 20-fold among non-homologous chromosomes. The most parsimonious model to reconcile these findings, particularly at human centromeres, is that CENP-A density within the subdomains that comprise the overall CEN chromatin domain varies between alpha satellite arrays on different chromosomes, including homologous chromosomes (Fig. 7a). In such a model, a preferred quantity of CENP-A is incorporated at every centromere, regardless of alpha satellite size, such that a core set of CENP-A nucleosomes is more important for centromere inheritance than the density or spacing [34, 35]. Indeed, previous studies of neocentromeres have shown that the sizes of CENP-A subdomains vary by an order of magnitude within the CEN chromatin domain [36, 37], supporting a model that density and spacing of CENP-A nucleosomes are not strictly uniform at a centromere.

CENP-A is important for building the centromere/kinetochore complex, and vertebrate centromeres in particular have an over-abundance of CENP-A [38], given that levels can be reduced by at least 80 % before widespread centromere defects are observed [39, 40]. Our data suggest that in order to maintain the proper amount of CENP-A on each alpha satellite array and to prevent replicated centromeres from dropping below a critical level, CENP-A nucleosomes are evenly diluted during S phase each cell cycle (Fig. 7b). We found that CENP-A dilution at each cell cycle appears to occur independently of the distribution pattern in the previous divisions. Therefore, while centromeres maintain equal amounts of CENP-A, there does not appear to be an obvious mechanism for counting and correcting CENP-A to a specific amount.

### A cycle-specific oscillating pattern of CENP-A loading defines the CEN chromatin and maintains centromere inheritance

Our results indicate that when new CENP-A is loaded each G1, it is placed within CENP-A chromatin in relation to old or existing CENP-A nucleosomes. The oscillating pattern of incorporation (i.e., CENP-A loaded near old CENP-A in consecutive cycles but in more similar positions to old CENP-A in alternating cycles) is consistent with the model of CENP-A incorporation at the location of H3.3 placeholder nucleosomes that are positioned into the gaps created during S phase distribution of CENP-A [16] (Fig. 7b). Such placeholder nucleosomes would allow CENP-A to be replenished at the same level while maintaining both a relatively constant number of nucleosomes and the genomic location of the centromere on a specific alpha satellite array. Deviation from this pattern of loading may have important implications for genome instability, particularly in cancers, where CENP-A molecules are often mistargeted or the CENP-A domain expands beyond its normal size [18, 41, 42]. Maintenance of CEN chromatin domain size and the position of new CENP-A loading on a fine scale might be controlled by chromatin remodelers or chaperones, such as ATRX and DAXX, that are able to impact H3.3 and CENP-A globally [43–45]. Finally, non-coding RNAs produced from centromere regions may serve as beacons for CENP-A loading or help to maintain the chromatin as a limited domain. A few studies have shown that transcripts are produced from human centromeres and that non-coding RNAs located within the CENP-A domain appear to recruit HJURP and CENP-A [46]. Whether a common RNA acts globally to control CEN chromatin at all chromosomes, or whether array-specific transcripts (SM McNulty and BA Sullivan, unpublished data) are linked to chromosome-specific CEN chromatin dynamics, however, remains to be explored.

### The importance and regulation of CENP-A chromatin boundaries

Our work has clearly shown that CENP-A assembles into a domain that maintains a finite size and is spatially confined to a portion of an alpha satellite DNA array. We did not observe substantial clusters of CENP-A outside of the primary CENP-A domain or large-scale movement of the CENP-A domain on alpha satellite DNA. These findings raise obvious questions regarding regulation of CEN chromatin domain size. We observed that CENP-A molecules were incorporated at a higher density near the boundaries of the CENP-A domain, suggesting an active mechanism for maintaining CENP-A boundaries. Several factors might control this process. CEN chromatin contains canonical histones that exhibit posttranslational modifications, such as methylation on lysine 4 (K4) and lysine 36 (K36). CEN chromatin domains lacking these modifications are unable to recruit new CENP-A [39], suggesting that a similarly finite region of H3K4me2/H3K36me within the centromere may help to regulate the loading of new CENP-A assembly within CEN chromatin and demarcate the far boundaries of the domain. However, centromere regions also contain heterochromatic histones, such as H3K9me3 and H3K27me, that are found at varying levels within the CEN domain, but are highly enriched in the pericentric regions flanking the CEN chromatin domain [10, 14, 47, 48]. Rather than CENP-A loading or H3K4me2/H3K36me exclusively regulating the CEN domain boundaries, the chromatin outside the region may delimit CENP-A chromatin. Indeed, when heterochromatin is depleted, the CENP-A domain increases in size and spreads beyond its original boundaries [18, 49, 50]. It is more likely that the maintenance of the CENP-A domain involves multiple players that work synergistically—heterochromatin and associated proteins to limit the size of the CEN chromatin domain, HJURP to bring pre-assembled CENP-A/H4 to the correct centromere region that has been licensed in telophase by Mis18 [35], incorporation of placeholder H3.3 during S phase by ATRX, DAXX, or other chaperones, modification of CENP-A, and/or methylation of H3K4/K36 to direct new CENP-A loading [16, 39]. Dissecting the hierarchy of such a complex pathway is a key area that remains to be explored in the context of CEN domain placement and maintenance on individual chromosomes.

## Conclusions

The assembly of CENP-A nucleosomes into the CEN chromatin domain that is faithfully maintained is important for genome and chromosome stability. Given the broad differences in alpha satellite DNA sequences and sizes at human chromosomes, how CENP-A loading and distribution differs among chromosomes has not been clear. Our analyses of nascent CENP-A incorporation and distribution emphasize the spectrum of CENP-A dynamics at human centromeres. The total amount and the distribution of nascent CENP-A to sister centromeres during S phase is largely equivalent among chromosomes. However, the genomic extent of CENP-A domain sizes varies among chromosomes in relation to the underlying size of specific alpha satellite arrays. This variation most likely represents differences in the density of CENP-A nucleosomes within subdomains in the CEN chromatin domain. New CENP-A is loaded in a cycle-specific oscillating pattern that keeps old and new CENP-A within a size-defined CEN chromatin domain while simultaneously maintaining the CEN chromatin domain at a fixed location on alpha satellite DNA. Our studies add a new dimension of information to the organization and plasticity of CENP-A chromatin and raise the possibility that diseases such as cancer disrupt normal regulation and dynamics of human centromeres.

## Methods

### Cell lines and culture

Human male fibroblast cell lines, HDF-XSN (PF; Fig. 3) and HT1080 (CF), were grown in MEM alpha supplemented with 10 % fetal bovine serum (FBS) and 1× antibiotic–antimycotic solution (Gibco). Male lymphoblast cell line LT690 (LCL) was grown in RPMI 1640 supplemented with 15 % FBS and 1× antibiotic–antimycotic (Gibco). A SNAP-CENP-A-HA construct, generously provided by Lars Jansen, was introduced into HT1080 cells, and clonal lines stably expressing the SNAP-CENP-A fusion protein were selected by blasticidin S (5 μg/mL). From these clones, a line demonstrating proper targeting of SNAP-CENP-A only at centromeres was selected and maintained in MEM alpha supplemented with 10 % fetal bovine serum (FBS), 1× antibiotic–antimycotic solution, and 5 μg/mL blasticidin S.

SNAP-CENP-A expression levels were determined by Western blotting with 40 μg of RIPA extracted protein samples from HT1080 and HT1080-SNAP-CENP-A cells after separation on SDS/polyacrylamide and transfer to PVDF using a semidry apparatus. Protein was detected with antibodies against CENP-A (custom rabbit polyclonal AP3497; Open Biosystems) and β-actin (Abcam, ab8227) and visualized using ECL. Quantification was performed in GeneTools software (Syngene), and HT1080-SNAP-CENP-A endogenous and SNAP-CENP-A levels were normalized to the HT1080 endogenous CENP-A band.

### Synchronization procedure

Synchronization in G1 phase for fibroblast cell lines was achieved by serum starvation in 0.5 % FBS supplemented MEM alpha for 48 h to arrest cells in G_0_, followed by return to complete medium with 10 μM EdU for 6 h to release cells into G1. Synchronization in G1 phase for the lymphoblast cell line was achieved by nocodazole treatment (50 ng/ml) for 15 h to arrest cells in mitosis, followed by three washes in 1× PBS, and release into complete medium with 10 μM EdU for 4 h to allow cells to enter G1. For all cell lines, synchronization at the G1/S phase boundary was accomplished by double thymidine block; cells were treated with 2 mM thymidine in complete medium for 17 h, washed three times in PBS, and released in complete medium for 8 h, followed by addition of thymidine to a final concentration of 2 mM for 17 h, with the addition of 10 μM EdU for the final 4 h. S phase synchronization was accomplished, in all cell lines, by double thymidine block (as described for G1/S synchronization), washed three times in 1× PBS, and then released into complete medium with 10 μM EdU for 6 h to allow cells to pass mid-S phase. EdU was added at 10 μM to label replicating DNA according to Click-iT EDU imaging kit (Invitrogen).

### SNAP quench and pulse labeling

HT1080 CENP-A-SNAP cells were synchronized by double thymidine block and then released (as described in S phase synchronization above). SNAP-tag activity was quenched by the addition of bromothenylpteridine (BTP) (SNAP-block; New England Biolabs, NEB) in complete growth medium for 30 min at 37 °C, washed twice with PBS and once with complete growth medium, and then re-incubated in complete growth medium for 30 min to allow excess compound to diffuse from cells. Cells were again washed twice in PBS and once with complete growth medium and then grown in complete growth media for an additional 6 h to allow synthesis of new CENP-A. Pulse labeling of cycle 1 was accomplished by incubation with 2 μM SNAP-Cell TMR-Star (NEB) or SNAP-Cell Oregon Green (NEB) for 30 min at 37 °C. Cells were washed, excess allowed to diffuse, and washes performed again, as done following SNAP-block quench. Cells were again treated with 2 mM thymidine in complete medium for 16 h to maintain synchronization. Over the following three days, the quench, pulse, and synchronization treatments were repeated for cycles 2, 3, and/or 4 as done for cycle 1. The day following cycle 4 treatment cells were again released from thymidine synchronization and used for either metaphase or chromatin fiber preparations.

### SNAP-CENP-A metaphase chromosome preparation and quantitative measurements

To measure the amount of total or nascent CENP-A on sister centromeres, CENP-A immunostaining (Abcam, ab13939) was performed on SNAP-CENP-A containing metaphase chromosomes that were cytospun onto slides. To analyze specific chromosomes, IF was followed by FISH with directly labeled (AF488-dUTP or AF594-dUTP, Invitrogen/Thermo Scientific) probes specific for D1Z7 (HSA1), D17Z1 (HSA17), DXZ1 (HSAX), and DYZ3 (HSAY) alpha satellite arrays. TIF files of deconvolved images were opened in ImageJ, and using a custom macro (available upon request), each fluorescent CENP-A signal in the metaphase spread was segmented after background subtraction. Integrated densities for each CENP-A fluorescent spot were exported to Excel, and CENP-A pairs representing sister centromeres were visually and manually matched. Integrated densities were also collected for each of the SNAP-CENP-A pools present at the centromere. CENP-A fluorescent signal pairs representing sister centromeres were added to arrive at the total CENP-A integrated density per centromere. The proportion of CENP-A divided between sisters was measured as a percentage of this total within each fluorescently labeled nascent CENP-A pool. Segregating units were calculated as described previously [23, 28]. The difference (δ) in fluorescence intensity and the sum (Σ) intensity of the sister centromeres were used to determine the fluorescence intensity per segregating unit (α) from the average δ^2^/Σ of all centromere pairs of the same experiment. The number of segregating units on each centromere was calculated as Σ/α. Segregating units for specific human chromosomes, identified by FISH with alpha satellite probes, were compared to the total across all chromosomes.

### IF-FISH on chromatin fibers

Extended chromatin fibers were prepared using published methods [13, 51]. Replication labeling on chromatin fibers from synchronized cells was performed by EdU detection according to Click-iT EdU imaging kit (Invitrogen). Following EdU detection, immunofluorescence with CENP-A antibodies (Abcam, ab13939) and FISH was performed as previously described [10, 13, 18]. PCR-generated X alpha satellite (DXZ1) and Y alpha satellite (DYZ3) probes for in situ hybridization were labeled with dUTPs conjugated to Alexa Fluor 488 or Alexa Fluor 568 (Molecular Probes, Thermo Scientific) [18]. Commercial directly labeled (red or green) alpha satellite FISH probes were used in some instances (Abbott Laboratories). A minimum of 12 chromatin fibers was analyzed for each cell line at each cell cycle phase.

### Microscopy

All images were acquired using an inverted Olympus IX-71 microscope controlled by the Deltavision Elite Imaging System (Applied Precision) equipped with a Photometric CoolSNAP HQ^2^ CCD camera. Images from individual metaphase spreads were collected at the same exposure time using a 40× objective. Deconvolved images (conservative ratio, 10 iterations) were projected and saved as Photoshop and TIF files. Fiber images were collected using the 100× objective, and those fibers extending through multiple fields of view were captured using the Panels option in the softWoRx Acquire 3D program and merged into single images using the ‘Stitch’ function. All images were exported for analysis into Adobe Photoshop and ImageJ.

### CENP-A domain size analysis

The ‘Measure Distances’ tool was used to calculate lengths of fluorescent signals representing euchromatic probes, alpha satellite probes or CENP-A immunostaining [10, 18]. CENP-A domain integrated density was measured from images collected at the same exposure time. CENP-A domain size was measured by comparing the length of CENP-A antibody staining (in micrometers) to the length of overlapping alpha satellite FISH probe. Alpha satellite FISH probe signal length represented total satellite array size that had been determined by pulsed field gel electrophoresis [18]. CENP-A domain size was calculated from the ratio of the length of CENP-A antibody signal over the total length of alpha satellite FISH signal [10, 18]. Analysis of variance between groups for the sizes of CENP-A domains across the cell cycle was assessed, with *p* values <0.01 considered statistically significant.

### SNAP-CENP-A chromatin fiber preparation and measurements

To measure the amount and placement of total or nascent CENP-A, extended chromatin fibers were prepared using published methods [13]. CENP-A immunostaining was performed on SNAP-CENP-A containing fibers as above. Images from individual fibers were collected at the same exposure time using a 100x objective. Deconvolved images (conservative ratio, 10 iterations) were projected and saved as Photoshop and TIF files. TIF images were opened in ImageJ, and using a custom macro, each CENP-A IF signal on the fiber was segmented after background subtraction. For each IF and SNAP-CENP-A channel, integrated densities for each CENP-A spot, and for each fiber as a whole, were exported to Excel. Each fiber was divided into four equal quarters (by length), the numbers of CENP-A spots were counted, and the total integrated density per quarter was summed. Co-localization of nascent CENP-A pools was measured using custom macros and the JACop plugin for ImageJ. Co-localization was assessed using both Pearson’s correlation coefficient and mass-particle coincidence. Differences in the degree of co-localization between pools of nascent CENP-A were determined using a Student’s *t* test. *p* values <0.01 were considered statistically significant.

## Additional files

10.1186/s13072-016-0071-7 Quantitation of CENP-A levels and proof-of-principle experiments to identify distinct pools of nascent CENP-A. (a) Western blotting to compare the amount of endogenous CENP-A to SNAP-CENP-A revealed that endogenous CENP-A levels were reduced by 26 %, and SNAP-CENP-A was present at similar levels to endogenous CENP-A within the HT1080-SNAP-CENP-A cell line. (b) Chromatin fibers from the HT1080 line expressing SNAP-CENP-A were immunostained for CENP-A followed by FISH with DXZ1. The size of the CENP-A domain remained ~ 35 %, indicating that SNAP-CENP-A expression did not appear to alter normal CENP-A dynamics. (c) Nascent CENP-A in synchronized cells was labeled in the first cell cycle with TMR-Star (red) and if cells proceeded to the next cell cycle, addition of SNAP-Oregon Green could detect nascent CENP-A from the previous cell cycle that had not been quenched or completely bound by TMR-Star. If cells were incubated after TMR-Star with 20 μM SNAP-block, nascent CENP-A that had not been completely saturated by TMR-Star was quenched and was not detectable with SNAP-Oregon Green. Scale bars are 15 μm. (d) The reverse order of the experiment in (c) was done to confirm that complete quenching could be achieved prior to detection with either SNAP-fluorophore of the subsequent nascent pool of CENP-A. Scale bars are 15 μm.
10.1186/s13072-016-0071-7 Visualization of the distribution of additional consecutive or alternate pools of nascent CENP-A. (a) Outline of multi-color SNAP-CENP-A labeling. (b) Representative results of visualizing distribution of nascent CENP-A loaded in cell cycles 1 and 4, cell cycles 2 and 4, and cell cycles 3 and 4. Scale bars are 5 μm.
10.1186/s13072-016-0071-7 Title: CENP-A fluorescence and segregating units per centromere. Description: Table showing CENP-A fluorescence (AFUs) and segregating units (SUs) for individual chromosomes analyzed in Figure 2, including statistical analysis.
10.1186/s13072-016-0071-7Title: CENP-A domain sizes through the cell cycle. Description: Table of DXZ1 and DYZ3 alpha satellite array sizes and CENP-A domain sizes, including percentage of total array, for three cell lines, including statistical analysis.
10.1186/s13072-016-0071-7Experimental strategy for isolation of G1, G1/S, and S phase chromatin fibers. (a) G1 (top), G1/S (middle), S (bottom) phase synchronization scheme for LCL and fibroblast lines using nocodazole block and release, serum starvation, and/or double thymidine blocks. The thymidine analog EdU was used in each experiment to differentiate between unreplicated and replicated centromeres. (b) Representative chromatin fibers stained with DAPI, EdU, and CENP-A antibodies (not shown), showing that the synchronization schemes were successful. Only EdU-negative fibers were analyzed for G1 and G1/S experiments; only EdU-positive fibers were analyzed for S phase experiments.
10.1186/s13072-016-0071-7 Spatial positioning of CENP-A loading on chromatin fibers using multi-color nascent protein labeling. (a) Experimental outline for detecting pools of SNAP-CENP-A loaded in different cell cycles. (b) Representative results of chromatin fibers stained for nascent CENP-A loaded in Cycle 1 versus Cycle 4 or consecutive Cycles 3 and 4. (c) Fluorescence line plots were used to visualize overlap between nascent CENP-A pools from various consecutive and alternate cell cycles.
10.1186/s13072-016-0071-7 Comparison of chromatin fibers in which multiple nascent fluorescent CENP-A pools were detected. (a) Representative chromatin fiber containing multiple pools of cycle-specific SNAP-CENP-A, showing that little nascent CENP-A was detected outside of the established CENP-A domain. (b) Chromatin fibers were divided into four equal quarters, and the intensity of total (T) and nascent (SNAP) CENP-A was measured between consecutive cycles (X, X + 1). (c) The same quantification was done as in (a) but for total (T) and nascent CENP-A fluorescence between alternate cell cycles (X, X + 2).
10.1186/s13072-016-0071-7 Title: Quantification of nascent CENP-A incorporation. Description: Table of nascent and total CENP-A signal on chromatin fibers, including percentage and average integrated density of fluorescent signals in specific quarters.

## Abbreviations

AFU: arbitrary fluorescence unit(s)
ATRX: alpha thalassemia/mental retardation, X-linked protein
BTP: bromothenylpteridine (SNAP-block)
CENP-A: centromere protein A
DAXX: death domain-associated protein
EdU: 5-ethynyl-2′-deoxyuridine
FISH: fluorescence in situ hybridization
HJURP: holliday junction recognition protein
SU: segregating units
