# Supplementary figures and images for: Inheritance of the CENP-A chromatin domain is spatially and temporally constrained at human centromeres
Source: Epigenetics Chromatin. 2016 May 31;9:20. doi: 10.1186/s13072-016-0071-7 (PMC4888493; doi:10.1186/s13072-016-0071-7)

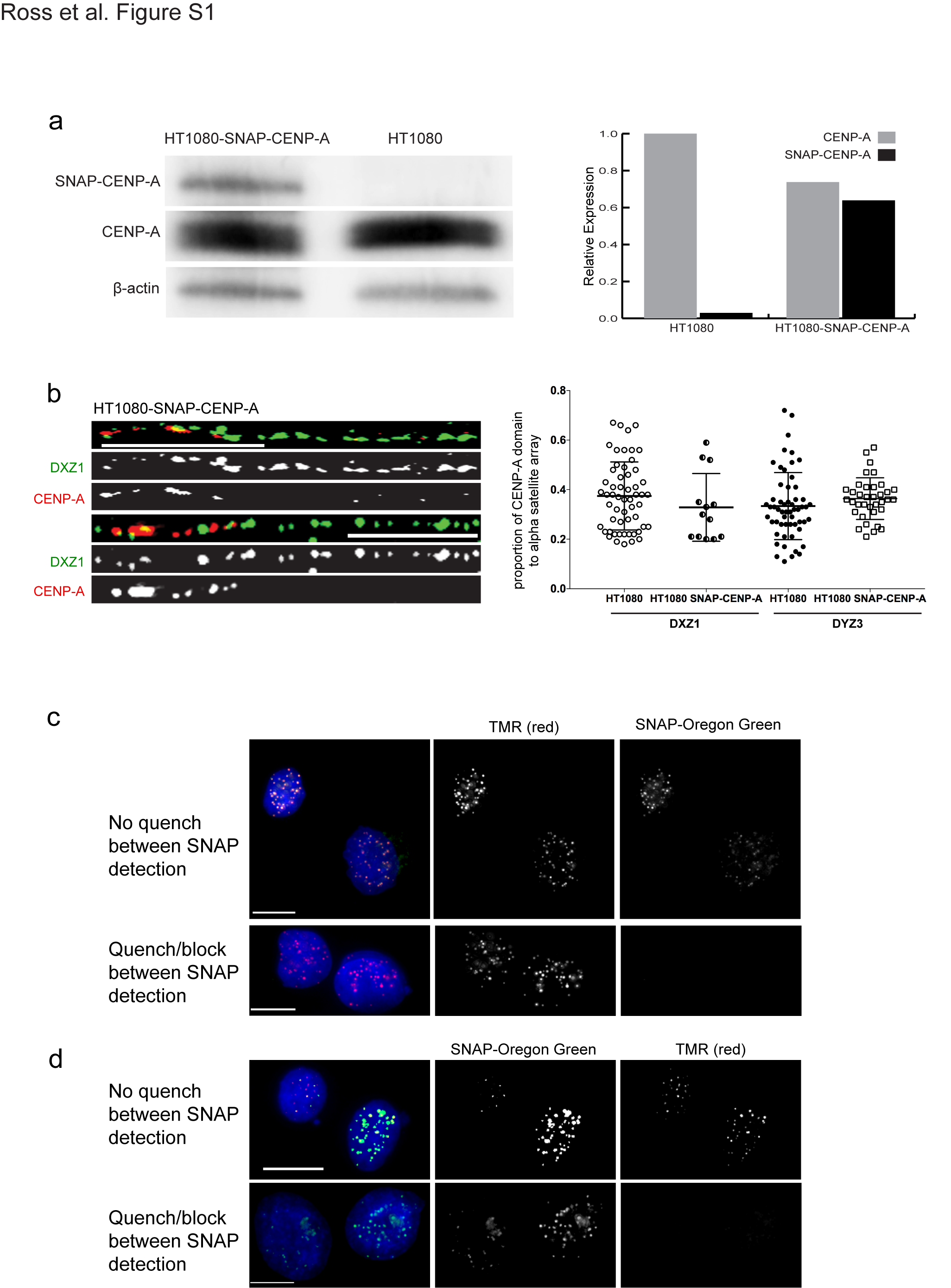

Supplement: Supplementary file 1 — 10.1186/s13072-016-0071-7 Quantitation of CENP-A levels and proof-of-principle experiments to identify distinct pools of nascent CENP-A. (a) Western blotting to compare the amount of endogenous CENP-A to SNAP-CENP-A revealed that endogenous CENP-A levels were reduced by 26 %, and SNAP-CENP-A was present at similar levels to endogenous CENP-A within the HT1080-SNAP-CENP-A cell line. (b) Chromatin fibers from the HT1080 line expressing SNAP-CENP-A were immunostained for CENP-A followed by FISH with DXZ1. The size of the CENP-A domain remained ~ 35 %, indicating that SNAP-CENP-A expression did not appear to alter normal CENP-A dynamics. (c) Nascent CENP-A in synchronized cells was labeled in the first cell cycle with TMR-Star (red) and if cells proceeded to the next cell cycle, addition of SNAP-Oregon Green could detect nascent CENP-A from the previous cell cycle that had not been quenched or completely bound by TMR-Star. If cells were incubated after TMR-Star with 20 μM SNAP-block, nascent CENP-A that had not been completely saturated by TMR-Star was quenched and was not detectable with SNAP-Oregon Green. Scale bars are 15 μm. (d) The reverse order of the experiment in (c) was done to confirm that complete quenching could be achieved prior to detection with either SNAP-fluorophore of the subsequent nascent pool of CENP-A. Scale bars are 15 μm. [file 13072_2016_71_MOESM1_ESM.tif]

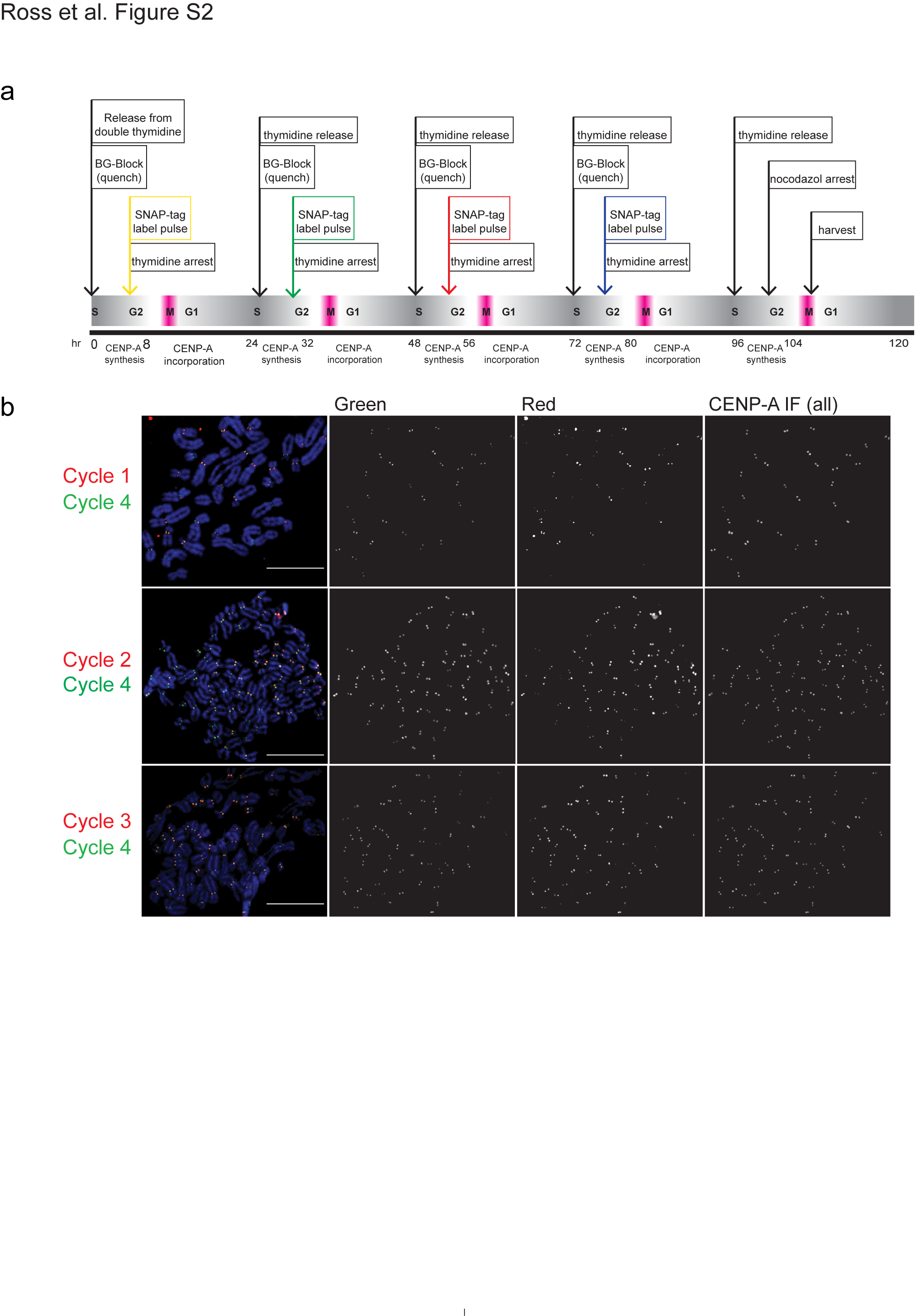

Supplement: Supplementary file 2 — 10.1186/s13072-016-0071-7 Visualization of the distribution of additional consecutive or alternate pools of nascent CENP-A. (a) Outline of multi-color SNAP-CENP-A labeling. (b) Representative results of visualizing distribution of nascent CENP-A loaded in cell cycles 1 and 4, cell cycles 2 and 4, and cell cycles 3 and 4. Scale bars are 5 μm. [file 13072_2016_71_MOESM2_ESM.tif]

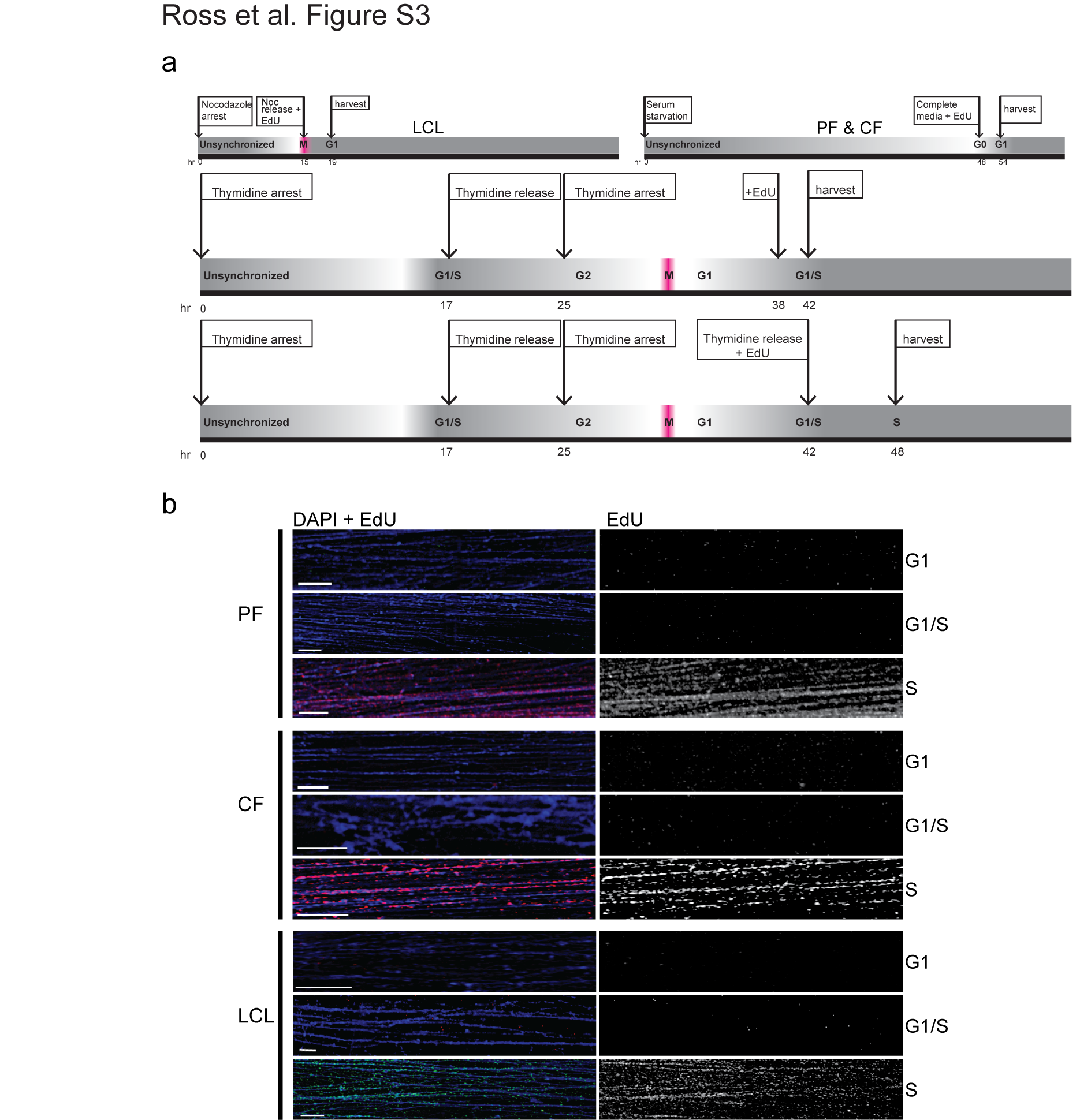

Supplement: Supplementary file 5 — 10.1186/s13072-016-0071-7Experimental strategy for isolation of G1, G1/S, and S phase chromatin fibers. (a) G1 (top), G1/S (middle), S (bottom) phase synchronization scheme for LCL and fibroblast lines using nocodazole block and release, serum starvation, and/or double thymidine blocks. The thymidine analog EdU was used in each experiment to differentiate between unreplicated and replicated centromeres. (b) Representative chromatin fibers stained with DAPI, EdU, and CENP-A antibodies (not shown), showing that the synchronization schemes were successful. Only EdU-negative fibers were analyzed for G1 and G1/S experiments; only EdU-positive fibers were analyzed for S phase experiments. [file 13072_2016_71_MOESM5_ESM.tif]

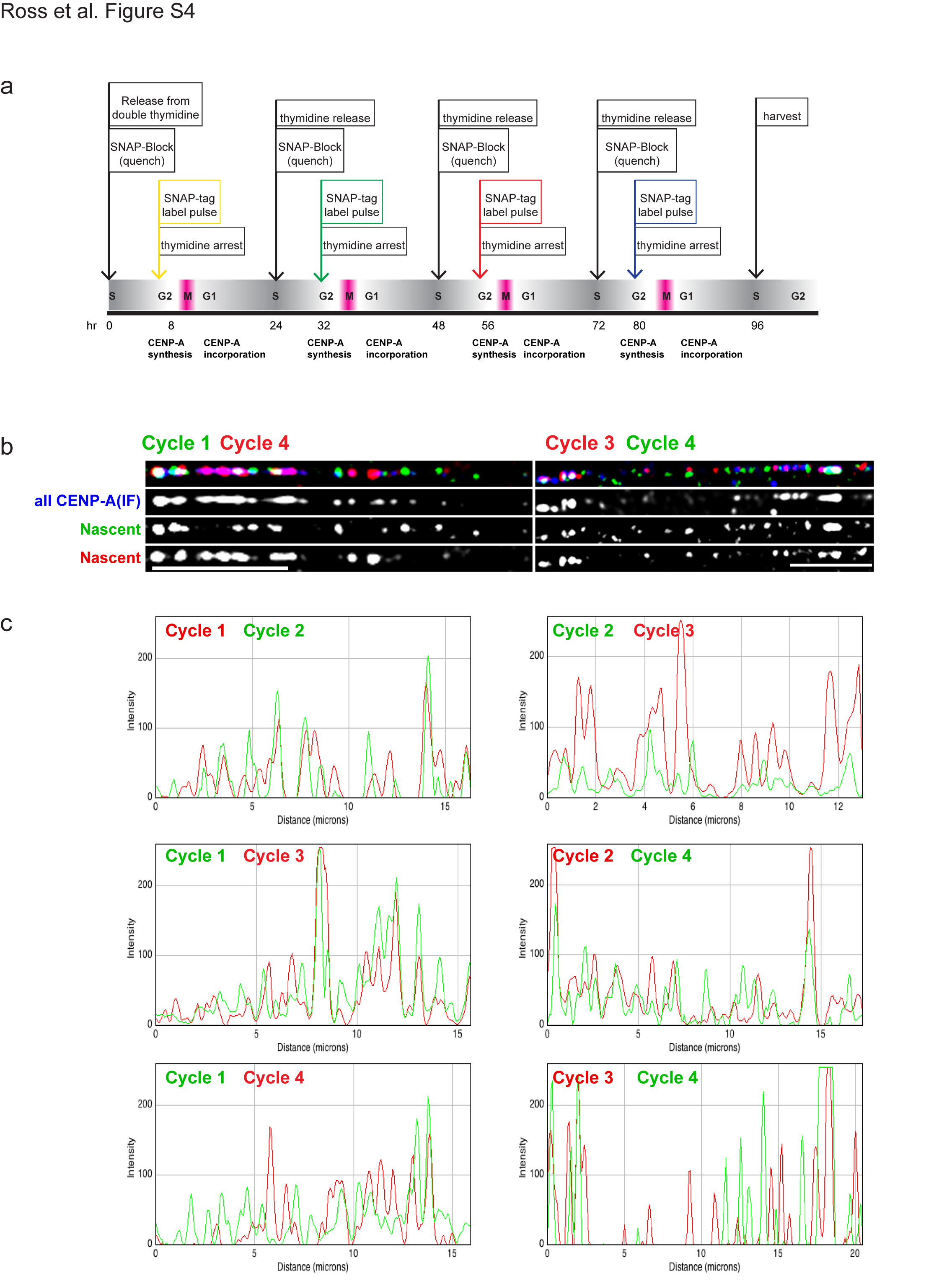

Supplement: Supplementary file 6 — 10.1186/s13072-016-0071-7 Spatial positioning of CENP-A loading on chromatin fibers using multi-color nascent protein labeling. (a) Experimental outline for detecting pools of SNAP-CENP-A loaded in different cell cycles. (b) Representative results of chromatin fibers stained for nascent CENP-A loaded in Cycle 1 versus Cycle 4 or consecutive Cycles 3 and 4. (c) Fluorescence line plots were used to visualize overlap between nascent CENP-A pools from various consecutive and alternate cell cycles. [file 13072_2016_71_MOESM6_ESM.tif]

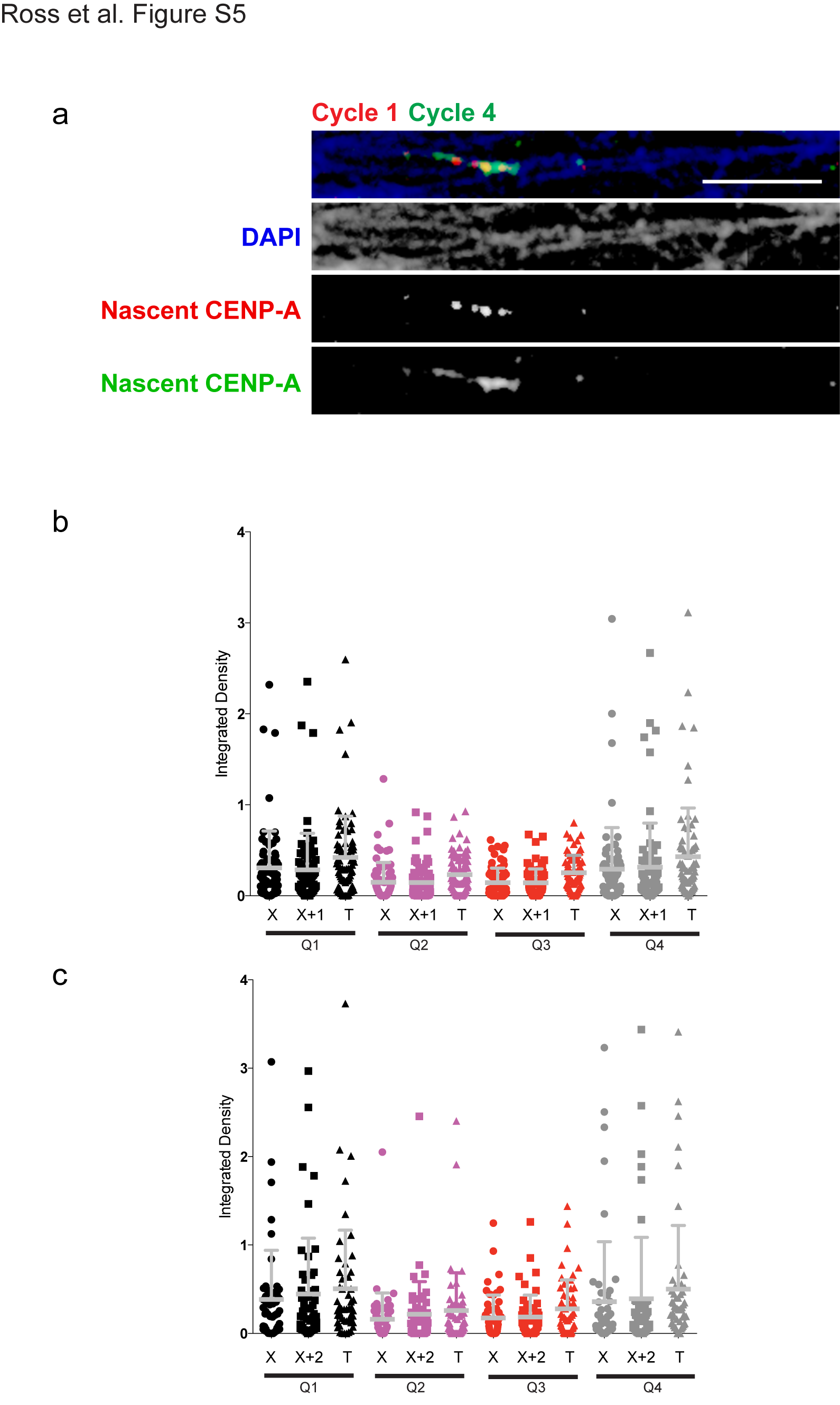

Supplement: Supplementary file 7 — 10.1186/s13072-016-0071-7 Comparison of chromatin fibers in which multiple nascent fluorescent CENP-A pools were detected. (a) Representative chromatin fiber containing multiple pools of cycle-specific SNAP-CENP-A, showing that little nascent CENP-A was detected outside of the established CENP-A domain. (b) Chromatin fibers were divided into four equal quarters, and the intensity of total (T) and nascent (SNAP) CENP-A was measured between consecutive cycles (X, X + 1). (c) The same quantification was done as in (a) but for total (T) and nascent CENP-A fluorescence between alternate cell cycles (X, X + 2). [file 13072_2016_71_MOESM7_ESM.tif]
