# Supplementary material for: Inheritance of the CENP-A chromatin domain is spatially and temporally constrained at human centromeres
Source: Epigenetics Chromatin. 2016 May 31;9:20. doi: 10.1186/s13072-016-0071-7 (PMC4888493; doi:10.1186/s13072-016-0071-7)
Supplement: Supplementary file 3 — 10.1186/s13072-016-0071-7 Title: CENP-A fluorescence and segregating units per centromere. Description: Table showing CENP-A fluorescence (AFUs) and segregating units (SUs) for individual chromosomes analyzed in Figure 2, including statistical analysis. [file 13072_2016_71_MOESM3_ESM.docx]

**Additional File 1. CENP-A fluorescence and segregating units per centromere**

|  | **CENP-A**  **Fluorescence (AFU)** | **Tukey HSD p value**  **(compared to HSAX)** | **Segregating Units** |
| --- | --- | --- | --- |
| **All Chromosomes** | 0.0273 (±0.0139) | N/A | 43.65 (±47.36) |
| **HSA1** | 0.0328 (±0.0173) | 0.0196 | 48.79 (±41.48) |
| **HSA17 (combined)** | 0.0350 (±0.0156) | 0.0003 | 45.03 (±37.46) |
| **HSA17**  **(homolog 1)** | 0.0390 (±0.0128) | N/A | 47.92 (±33.32) |
| **HSA17**  **(homolog 2)** | 0.0226 (±0.0118) | N/A | 32.71 (±27.07) |
| **HSAX** | 0.0211 (±0.0111) | N/A | 27.60 (±18.38) |
| **HSAY** | 0.0286 (±0.0113) | 0.1766 | 46.31 (±30.72) |
| **Between chromosome ANOVA** | | F(3,176) = 3.546,  p value = 0.0004 |  |

CENP-A fluorescence was measured on metaphase chromosomes of at all centromeres and specifically on chromosomes HSA1, HSA17, HSAX, and HSAY. ANOVA results signify that all chromosomes are not equivalent in CENP-A fluorescence and Tukey HSD reveals significant differences with HSAX compared to both HSA1 and HSA17 (while all other pairwise comparisons are non-significant). Calculations of segregating units, from stochastic fluctuations of CENP-A fluorescence between sister centromeres, had similar results across all chromosomes.
