# Supplementary material for: Inheritance of the CENP-A chromatin domain is spatially and temporally constrained at human centromeres
Source: Epigenetics Chromatin. 2016 May 31;9:20. doi: 10.1186/s13072-016-0071-7 (PMC4888493; doi:10.1186/s13072-016-0071-7)
Supplement: Supplementary file 4 — 10.1186/s13072-016-0071-7Title: CENP-A domain sizes through the cell cycle. Description: Table of DXZ1 and DYZ3 alpha satellite array sizes and CENP-A domain sizes, including percentage of total array, for three cell lines, including statistical analysis. [file 13072_2016_71_MOESM4_ESM.docx]

**Additional File 2. CENP-A domain sizes through cell cycle**

|  |  | **Lymphoblast line (LCL)** | **Cancer fibroblast (CF)** | **Primary fibroblast (PF)** |
| --- | --- | --- | --- | --- |
| **Alpha satellite size (Mb)** | DXZ1 | 1.5 | 3.0 | 4.2 |
| **CENP-A domain size (Mb)** | G1 | 0.47 (31%) | 1.02 (34%) | 1.32 (32%) |
| **(% of array)** | G1/S | 0.48 (32%) | 1.09 (36%) | 1.60 (38%) |
|  | S | 0.46 (30%) | 1.13 (38%) | 1.46 (35%) |
| **Between cell cycle ANOVA** |  | F(2,51) = 0.036, *p* value = 0.964 | F(2,50) = 0.421,  *p* value = 0.659 | F(2,50) = 2.363,  *p* value = 0.105 |
| **Alpha satellite size (Mb)** | DYZ3 | 0.37 | 1.1 | 1.1 |
| **CENP-A domain size (Mb)** | G1 | 0.13 (34%) | 0.33 (30%) | 0.37 (34%) |
|  | G1/S | 0.11 (30%) | 0.38 (35%) | 0.40 (38%) |
|  | S | 0.12 (32%) | 0.37 (34%) | 0.33 (30%) |
| **Between cell cycle phase ANOVA** |  | F(2,27) = 0.534,  *p* value = 0.592 | F(2,53) = 0.753,  *p* value = 0.476 | F(2,53) = 1.489,  *p* value = 0.235 |

Chromatin fiber analysis revealed that CENP-A domain sizes on chromosomes X (DXZ1) and Y (DYZ3) remained constant throughout the cell cycle, with no significant differences between cycle stages. In each line, at all time points, the CENP-A domain occupied approximately one-third of the alpha satellite array (size previously determined).
