# Supplementary material for: Inheritance of the CENP-A chromatin domain is spatially and temporally constrained at human centromeres
Source: Epigenetics Chromatin. 2016 May 31;9:20. doi: 10.1186/s13072-016-0071-7 (PMC4888493; doi:10.1186/s13072-016-0071-7)
Supplement: Supplementary file 8 — 10.1186/s13072-016-0071-7 Title: Quantification of nascent CENP-A incorporation. Description: Table of nascent and total CENP-A signal on chromatin fibers, including percentage and average integrated density of fluorescent signals in specific quarters. [file 13072_2016_71_MOESM8_ESM.docx]

**Additional File 3. Quantitation of nascent CENP-A incorporation**

|  | **all CENP-A (IF)** | **SNAP-CENP-A-TMR** | | **SNAP-CENP-A-Oregon** |
| --- | --- | --- | --- | --- |
| ***# of Quarters*** | ***Percent of fibers with CENP-A signal **** | | | |
| 4 (all) | 72.12 | 65.45 | 59.39 | |
| 3 | 23.63 | 25.45 | 27.27 | |
| 2 | 4.24 | 9.09 | 12.73 | |
| 1 | 0.00 | 0.00 | 0.61 | |
| ***Specific Quarter*** | ***Average Integrated Density of Fibers ***** | | | |
| 1 | 0.431 | 0.370 | 0.288 | |
| 2 | 0.234 | 0.197 | 0.139 | |
| 3 | 0.247 | 0.193 | 0.122 | |
| 4 | 0.473 | 0.405 | 0.345 | |
| ***CENP-A IF Normalized*** | ***Average Integrated Density of Fibers ****** | | | |
| Outer Quarters | N/A | 1.372 | 1.061 | |
| Inner Quarters | N/A | 0.995 | 0.777 | |
| *p* value | N/A | <0.0001 | <0.0001 | |

The CENP-A domain on chromatin fibers containing SNAP-CENP-A labeled in two cell cycles were divided into four quarters (of equal length) and analyzed for nascent CENP-A placement.

* # of Quarters specifies how many quarters were occupied by all, or nascent, CENP-A, i.e. 64.45% of fibers had SNAP-CENP-A-TMR in all four quarters while 0% were restricted to only 1 quarter.

** Specific quarter refers to how much CENP-A was present (measured in AFU) within each quarter.

*** When normalized to total CENP-A nascent CENP-A continued to have a higher integrated density at outer quarters, indicating an overabundance of nascent CENP-A.
